# Variational inference for detecting differential translation in ribosome profiling studies

**DOI:** 10.3389/fgene.2023.1178508

**Published:** 2023-06-23

**Authors:** David C. Walker, Zachary R. Lozier, Ran Bi, Pulkit Kanodia, W. Allen Miller, Peng Liu

**Affiliations:** ^1^ Department of Statistics, Iowa State University, Ames, IA, United States; ^2^ Department of Plant Pathology, Entomology and Microbiology, Iowa State University, Ames, IA, United States

**Keywords:** riboVI, variational inference, variational Bayes (VB), ribosome profiling (Ribo-seq), next-generation sequencing, RNA sequencing (RNA-seq)

## Abstract

Translational efficiency change is an important mechanism for regulating protein synthesis. Experiments with paired ribosome profiling (Ribo-seq) and mRNA-sequencing (RNA-seq) allow the study of translational efficiency by simultaneously quantifying the abundances of total transcripts and those that are being actively translated. Existing methods for Ribo-seq data analysis either ignore the pairing structure in the experimental design or treat the paired samples as fixed effects instead of random effects. To address these issues, we propose a hierarchical Bayesian generalized linear mixed effects model which incorporates a random effect for the paired samples according to the experimental design. We provide an analytical software tool, “riboVI,” that uses a novel variational Bayesian algorithm to fit our model in an efficient way. Simulation studies demonstrate that “riboVI” outperforms existing methods in terms of both ranking differentially translated genes and controlling false discovery rate. We also analyzed data from a real ribosome profiling experiment, which provided new biological insight into virus-host interactions by revealing changes in hormone signaling and regulation of signal transduction not detected by other Ribo-seq data analysis tools.

## 1 Introduction

Translational control is a key component in the regulation of gene expression. It is critical for growth, maintaining homeostasis, and responding to stress (Hershey et al., 2012). Aberrant regulation of protein synthesis in humans is implicated in serious diseases such as Parkinson’s, Alzheimer’s, and cancer (Holland, 2004; Bottley and Kondrashov, 2013; Ruggero, 2013; Taymans et al., 2015). Plant and animal viruses hijack the host’s translation machinery to translate viral RNA and make cellular conditions conducive to viral replication (Reid et al., 2018; Stern-Ginossar et al., 2019). For these reasons, understanding translational control mechanisms and the role of translationally regulated proteins is crucial to understanding cellular processes and disease.

High-throughput techniques such as RNA sequencing (RNA-seq) have been widely used to assess gene expression. These techniques use total mRNA levels as a proxy for protein abundance. However, protein levels do not always correlate closely with total mRNA (De Godoy et al., 2008) because measurements of total mRNA alone do not account for mechanisms of translational regulation that operate upon mRNA post-transcriptionally (Baek et al., 2008; Hinnebusch et al., 2016). The rate of protein synthesis is a better predictor of protein abundance than measurements of total mRNA (Ingolia et al., 2009), and methods that quantify both translation rate and total mRNA provide a more accurate assessment of gene expression. Ribosome profiling (Ribo-seq) is one such method. In addition to providing a quantitative measure of translation efficiency, Ribo-seq maps ribosomes to mRNAs at single nucleotide resolution, allowing the identification of precise sites of translational control, such as ribosome pause sites required for protein folding or shifts between initiation-limited and elongation-limited translation of a specific mRNA (Flanagan et al., 2022b; a).

Ribo-seq entails sequencing of ribosome-protected fragments (RPFs): short tracts of mRNA inside the translating ribosome and thus protected from ribonuclease digestion. RPFs are sequenced in parallel with total mRNA from the same samples to compare levels of translation between different treatments after controlling for transcript abundance. More specifically, both Ribo-seq and RNA-seq are performed on the same initial translation-arrested lysate; part of the lysate is digested with RNase to degrade all mRNA not protected by a translating ribosome and is then sequenced to produce the RPF counts; another part of the lysate is sequenced without being digested to produce the total mRNA transcript counts. Any sample-specific biological or technical effects incurred prior to RNase digestion will affect both RPF and mRNA. It is therefore desirable to model a shared random effect for RPF and mRNA counts from the same sample.

A common goal in the analysis of Ribo-seq data is to identify genes that exhibit differential translational efficiency across conditions, i.e., differentially translated genes (DTGs). The data matrix to be analyzed consists of both RPF counts and transcript counts for all genes in each sample (Table 1). Several methods have been proposed or adopted to analyze such a count data matrix and detect DTGs. We group these methods into two categories: i) methods adopted from differential expression analysis of RNA-seq data, including edgeR (Chen et al., 2014), DESeq2 (Love et al., 2014), and baySeq (Hardcastle and Kelly, 2010); ii) methods originally proposed for detection of DTGs in Ribo-seq data, including xtail (Xiao et al., 2016), RiboDiff (Zhong et al., 2017), and babel (Olshen et al., 2013).

**TABLE 1:** Example table of paired RPF/mRNA read counts; a subset of genes and samples from the Arabidopsis data described in Section 2.4

	Treatment 1						Treatment 2					
	Sample 1		Sample 2		Sample 3		Sample 1		Sample 2		Sample 3	
Gene	RPF	mRNA	RPF	mRNA	RPF	mRNA	RPF	mRNA	RPF	mRNA	RPF	mRNA
AT1G18560	49	76	87	61	131	71	126	430	113	326	168	330
AT1G63420	83	220	130	182	250	232	104	519	93	472	161	351
AT5G62950	24	69	41	40	121	60	56	279	66	265	88	197
AT2G04780	883	1244	1078	883	1834	860	824	1864	401	2123	897	1343
AT2G39220	106	221	144	142	365	136	238	805	158	1131	295	596
AT4G23240	37	54	39	30	87	26	108	149	87	154	168	194

DESeq2 and edgeR use a conceptually similar model: a generalized linear model (GLM) based on the negative binomial distribution with a log link function, where the differential translational efficiency is represented by an interaction term in their model. These methods do not accommodate random effects. Hence, the pairing structure is handled by having a fixed effect for each sample. However, it is more natural to think of the sample effects as random because we are interested in generalizing the conclusion to the whole target population, not just the specific samples in the study. Also, the random effects model tends to produce better estimates when the number of observations per sample is small (Clark and Linzer, 2015), which is the case for Ribo-seq studies.

BaySeq uses an empirical Bayes method for paired RNA-seq experiments that models counts from paired samples as beta-binomial distributed and estimates prior distributions for mean and dispersion parameters from the data using the maximum likelihood method. Under this model, DTGs are identified based on the posterior probability that there is a difference in ratio for paired counts between treatment conditions. While able to explicitly model the paired structure of Ribo-seq, baySeq does not produce effect size estimates and makes it difficult to represent and interpret results for complex designs.

RiboDiff implements a method similar to edgeR or DESeq2, but separately estimates the dispersion trend with respect to the mean count for total mRNA and RPF, respectively. Xtail uses an ad-hoc approach to adapt DESeq2 to Ribo-seq: rather than fitting a GLM with a shared parameter for mRNA and RPF from the same sample, it fits models to the mRNA and RPF counts separately, and then compares the estimated log fold change parameters using a simulation technique motivated by empirical Bayes. Both RiboDiff and xtail cannot accommodate designs with more than two treatment conditions or a block effect for paired counts.

Babel constructs a *p*-value for each pair of mRNA and RPF counts, i.e., one *p*-value per gene per sample, which is intended to test the null hypothesis that the RPF count is as expected given the mRNA count. Babel then uses an ad-hoc approach to combine the set of *p*-values across samples into a single *p*-value for each gene for the null hypothesis that the gene does not exhibit differential translational efficiency. Babel cannot represent experimental designs with more than two treatment conditions and does not estimate an effect size for translational efficiency.

We propose a fully Bayesian hierarchical GLMM (generalized linear mixed model) for detecting differential translational efficiency in Ribo-seq experiments and present a variational Bayesian algorithm and software tool, riboVI, to fit the model. A variational Bayesian algorithm is a computationally efficient method for approximating Bayesian posteriors in order to conduct inference, and can be particularly beneficial in high-dimensional applications like the analysis of Ribo-seq. Variational Bayesian approaches have been used successfully in other applications to high-throughput sequencing data (Teschendorff et al., 2005; Zhao et al., 2021).

Our proposed variational Bayesian method improves upon available methods for detecting DTGs by addressing the limitations described above. First, our modeling approach is tailored to Ribo-seq experiments, explicitly models the parameter of interest regarding translational efficiency, and uses the posterior distribution to detect DTGs. Second, our Bayesian method naturally borrows information across all genes and improves the performance of statistical inference in settings with a small number of replicates but a large number of variables. Information borrowing across genes occurs through the hierarchical Bayesian model structure, where gene-specific parameters share appropriate prior distributions. More specifically, the posterior estimation of the gene-specific translational efficiency utilizes information both from data on this specific gene and the prior distribution shared by all genes. Third, our model can represent the pairing between RPF and total transcript counts as a shared random effect. Fourth, our method more accurately classifies genes that exhibit differential translational efficiency and exhibits superior false discovery rate control over other methods.

In the remaining sections of this paper, we specify our model and outline our variational Bayesian algorithm to fit the model to Ribo-seq data. We then describe the simulation studies and real data analysis that we conducted to compare the performance of our method to existing methods. Finally, we discuss remaining areas for improvement and future research.

## 2 Methods

Ribo-seq data can be visualized as a table of counts, with rows for genes and columns for samples (Table 1). RPF read counts are used in conjunction with mRNA read counts from the same set of samples to identify genes that are translated at different rates (relative to the abundance of transcripts) between treatment conditions. This can be called differential translational efficiency analysis.

Because paired RPF and mRNA counts are derived from the same biological sample and undergo several technical preparation steps together before being processed in parallel, they are expected to share some random biological and technical effects in common. Hence, incorporating such random effects is important to appropriately analyze Ribo-seq data from paired experimental designs.

In this section, we first describe our GLMM model that incorporates a random effect for the pairing, present our variational Bayesian algorithm for obtaining posterior distributions, and then describe how to control multiple testing error. Finally, we describe the experimental procedures for a Ribo-seq experiment with *Arabidopsis* plants.

### 2.1 Model

For *g* = 1, *…* , *G*, *i* = 1, *…* , *N*, and *j* = 1, 2, let *y*_*gij*_ represent the read count for gene *g*, sample *i*, and preparation *j*, where *j* = 1 corresponds to RNA-seq count, and *j* = 2 corresponds to Ribo-seq count (RPF). We use the following hierarchical model for the data:

$$\begin{array}{cc} y_{\mathrm{gij}} & ∼\text{Pois}\left(\lambda_{\mathrm{gij}}\right),\\ \text{log}\left(\lambda_{\mathrm{gij}}\right) & =x_{ij}^{T}\beta_{g}+u_{gi},\\ u_{gi} & \overset{iid}{∼}\text{N}\left(0,\sigma_{u}^{2}\right). \end{array}$$ (1)

Model (1) is a Poisson-log-normal hierarchical model that can be viewed as an approximation to the Poisson-gamma model, whose marginal distribution is the negative binomial model that has been widely used to model RNA-seq data. Instead of using a negative binomial model directly, we propose to use Model (1) because it allows incorporating effects due to experimental design straightforwardly, and the parameters are easy to understand and interpret.

More specifically, the Poisson mean *λ*_*gij*_ is modeled by the fixed effects ***β***_*g*_ due to treatment and preparation (RPF or RNA-seq) and random effects *u*_*gi*_, where *u*_*gi*_ explicitly represents the sample-specific random effects shared by each pair of RNA-seq and RPF counts. Through *u*_*gi*_, our model handles variation due to biological replicates and accommodates overdispersion compared to a simple Poisson distribution. For an experiment with two treatment conditions, the parameter vector ***β***_*g*_ has four elements and we denote them as (*β*_*g*0_, *β*_*g*1_, *β*_*g*2_ and, *β*_*g*3_). We let *β*_*g*0_ be the mean expression level (on the log-scale) for RNA-seq data under the control condition; *β*_*g*0_ + *β*_*g*1_ be the mean expression level for RNA-seq data under treatment; *β*_*g*0_ + *β*_*g*2_ be the mean level for RPF under control condition; and *β*_*g*0_ + *β*_*g*1_ + *β*_*g*2_ + *β*_*g*3_ be the mean level for RPF under treatment. Accordingly, we have $x_{ij}={\left(1,\text{I}\left(i\in T_{2}\right),\;\text{I}\left(j=2\right),\;\text{I}\left(j=2\right)\text{I}\left(i\in T_{2}\right)\right)}^{T}$ representing the experimental design, where I (⋅) is an indicator function. Group means for the 2 × 2 design are shown in Table 2.

**TABLE 2:** Table of means on the log-scale for the 2 × 2 design for Model 2.1

Preparation	Control	Treatment
RNA-Seq	*β*_0_	*β*_0_ + *β*_1_
RPF	*β*_0_ + *β*_2_	*β*_0_ + *β*_1_ + *β*_2_ + *β*_3_

With this parameterization, translational efficiency is modeled by *β*_*g*2_ = (*β*_*g*0_ + *β*_*g*2_) − *β*_*g*0_ for the control group and *β*_*g*2_ + *β*_*g*3_ = (*β*_*g*0_ + *β*_*g*1_ + *β*_*g*2_ + *β*_*g*3_) − (*β*_*g*0_ + *β*_*g*1_) for the treatment group. The log-scale difference in translational efficiency between treatment and control is therefore *β*_*g*3_. In other words, the parameter *β*_*g*3_ corresponds to the interaction effect between treatment and preparation (Ribo-seq and RNA-seq), and hence it represents differential translational efficiency between treatments for gene *g*. With this parameterization, to determine differential translational efficiency amounts to testing $H_{0}^{g}:\beta_{g3}=0$ against *H*_*A*_: *β*_*g*3_ ≠ 0 for each gene *g*.

In an experiment, some genes exhibit differential translational efficiency while other genes do not. Hence *β*_*g*3_ can be modeled by a mixture of a continuous distribution and a point-mass at zero across all genes. We use such a mixture distribution as the prior distribution for *β*_*g*3_:

$$\begin{array}{cc} \beta_{g3} & =\left(1-D_{g}\right)W_{g},\\ D_{g} & \overset{iid}{∼}\text{Bern}\left(\pi_{0}\right),\\ W_{g} & \overset{iid}{∼}\text{N}\left(\mu_{\beta_{3}},\sigma_{\beta_{3}}^{2}\right). \end{array}$$ (2)

In model (2), Bern(*π*_0_) represents a Bernoulli distribution with parameter (*π*_0_). The null hypothesis that *β*_*g*3_ = 0 is then equivalent to the following hypothesis for each gene.

$$H_{0}^{g}:D_{g}=1$$ (3)

We use Gaussian distributions $\text{N}\left(\mu_{\beta_{p}},\sigma_{\beta_{p}}^{2}\right)$ as prior distributions for the remaining *β*_*gp*_ parameters (*p* = 0, 1, 2) because we are not concerned with testing other sharp nulls. This also simplifies the algorithm required to fit the model. If testing for differential expression based on RNA-seq is also of interest, we could use a mixture distribution of Gaussian and point mass at zero as the prior distribution for the parameter *β*_*g*1_.

We use conjugate prior distributions for the hyperparameters:

$$\begin{array}{cc} \sigma_{u}^{2} & ∼\text{IG}\left(\alpha_{u},\gamma_{u}\right),\\ \sigma_{\beta p}^{2} & \overset{ind}{∼}\text{IG}\left(\alpha_{\beta p},\gamma_{\beta p}\right),\\ \mu_{\beta_{p}} & ∼\text{N}\left(0,\sigma_{\mu }^{2}\right),p=0,\ldots ,3. \end{array}$$ (4)

### 2.2 Variational Bayesian algorithm

Since the Bayesian posterior for our model is not analytically tractable, we propose a novel variational inference algorithm to approximate the posterior in order to carry out inference. Our algorithm extends an existing algorithm for variational inference with Bayesian generalized linear mixed models to include our model’s hierarchical structure and the mixture prior for *β*_*g*3_. In this subsection, we give a brief introduction to variational inference in general, then describe the algorithm we use to fit our model specified in Section 2.1.

Variational inference is an alternative to sampling-based inference like Markov chain Monte Carlo (MCMC) for approximating an intractable posterior in Bayesian methods. Variational inference finds a close approximation to the true posterior by numerical optimization. This is achieved by first defining a family of distributions of which the variational posterior *q*(***θ***) will be a member and then minimizing the Kullbeck-Liebler divergence to the true posterior *p*(***θ***): KL(*q*(***θ***)‖*p* (***θ*** | ***y***)), where ***θ*** represents the set of model parameters and ***y*** represents the data.

Often the family of the variational posterior is defined by imposing what is called a mean-field assumption: the variational posterior must be factorizable. This enables the derivation of closed-form expressions for the optimal values of the individual factors given the other factors. These expressions form the basis of an iterative algorithm. During every iteration, each factor is updated to its optimal value given the current values of the other factors before recalculating the Kullbeck-Liebler divergence. The algorithm terminates when the Kullbeck-Liebler divergence converges to a stable value.

Our algorithm follows this basic structure, and is an extension and synthesis of work done by Tan and Nott (2013) and Wand (2014) in order to accommodate the hierarchical structure of our model and the mixture prior that is key to identifying differentially translated genes, the scientific questions of interest in Ribo-seq data analysis.

We define the distributional family of the variational posterior with the following mean-field assumption: the distribution *q*(***θ***) must satisfy Eq. 7, where $\beta_{g}^{*}=\left(\beta_{g0},\beta_{g1},\beta_{g2}\right)$ . Referring to our model specified in Section 2.1, ***D*** represents the set ${\left\{D_{g}\right\}}_{g\leq G}$ and ***ϕ*** represents the set of all hierarchical mean and variance parameters.

$$D={\left\{D_{g}\right\}}_{g\leq G}$$ (5)

$$\phi =\left\{\mu_{\beta },\sigma_{u}^{2},{\left\{\sigma_{\beta_{p}}^{2}\right\}}_{\forall p},\pi_{0}\right\}$$ (6)

$$q\left(\theta \right)=q_{\phi }\left(\phi \right)q_{D}\left(D\right)\underset{g\leq G}{\prod }q_{g}\left(\beta_{g}^{*},u_{g},W_{g}\right)$$ (7)

For readability we will suppress the subscripts on the factors of the variational distribution wherever context makes it clear which variational factor is referred to; e.g., *q*(***ϕ***) instead of *q*_*ϕ*_(***ϕ***). We use *q*_*m*_ as shorthand for the variational factor associated with parameter ***m***, and $q_{m}^{*}$ as shorthand for the optimal value of this variational factor.

We will first describe the method for updating the variational distribution for parameters where the mean-field assumption alone implies that the optimal value of the variational distribution has the same functional form as the prior for that parameter, which we call conjugate updates. Next, we describe the method of updating the variational distribution for parameters where this is not the case, called non-conjugate updates.

For *q*(***D***) and *q*(***ϕ***) in the factorized distribution in Eq. 7, we can derive the expression for the optimal value with respect to the other factors by simplifying Eq. 8. In Eq. 8, $q_{m}^{*}$ can represent the optimal value for either *q*(***D***) or *q*(***ϕ***), ***m*** can represent either ***D*** or ***ϕ***, and $\underset{-m}{\text{E}}\left(\cdot \right)$ represents the expectation taken with respect to the current values of all other factors excluding ***m*** in Eq. 7 at each iteration of the algorithm. Because these parameters have exponential family priors and are conjugate to all the neighboring distributions in the factor graph of the full join distribution for Model (1), their optimal values will have the same functional form as their priors (Bishop, 2006; Blei et al., 2017). The derivations of update expressions for *q*(***D***) and *q*(***ϕ***) are given in the supplementary material.

$$\text{ln}\left(q_{m}^{*}\right)=\underset{-m}{\text{E}}\left[\text{ln}\left(p\left(y,\theta \right)\right)\right]+\text{constant}$$ (8)

$$q_{m}^{*}=\frac{\text{exp}\left(\underset{-m}{\text{E}}\left[\text{ln}\left(p\right.\left(y,\theta \right)\right]\right)}{\int \text{exp}\left(\underset{-m}{\text{E}}\left[\text{ln}\left(p\right.\left(y,\theta \right)\right]\right)dm}$$ (9)

Having outlined the method for conjugate updates, we turn to the non-conjugate updates. For the factors of $\underset{g\leq G}{\prod }q\left(\beta_{g}^{*},u_{g},W_{g}\right)$ , i.e., for each $q\left(\beta_{g}^{*},u_{g},W_{g}\right)$ , we do not arrive at a recognizable distribution by simplifying Eqs 8, 9 because the priors for these parameters are not conjugate to the Poisson distribution we use to model the data. To arrive at an update expression for the distributions of these parameters, we further specify an exponential family form for the variational distribution $q\left(\beta_{g}^{*},u_{g},W_{g}\right)$ , shown in Eq.10. This approach is called non-conjugate message passing, and is described in detail in Knowles and Minka (2011) and Tan and Nott (2013). Using the exponential family specification, we are able to derive an expression for the optimal value of the natural parameter of the exponential family distribution. This is shown in Eq. 11, where $\lambda_{g}$ is the natural parameter for the exponential family and $V\left(\lambda_{g}\right)$ is the variance-covariance matrix of the sufficient statistic *t* ( $\kappa_{g}$ ). The expansion and simplification required to apply Eq. 11 in our algorithm are given in the supplementary material.

$$\begin{array}{cc} \kappa_{g}: & ={\left(\beta_{g}^{*},W_{g},u_{g}\right)}^{T}\\ q\left(\kappa_{g}\right) & =\text{exp}\left(\lambda_{g}^{T}t\left(\kappa_{g}\right)-h\left(\lambda_{g}\right)\right) \end{array}$$ (10)

$$\lambda_{g}\leftarrow V{\left(\lambda_{g}\right)}^{-1}\frac{\partial \underset{-m}{\text{E}}\left[\text{ln}\left(p\right.\left(y,\theta \right)\right]}{\partial \lambda_{g}}$$ (11)

By combining the conjugate and non-conjugate updates, we arrive at our complete iterative algorithm, summarized in Algorithm 1. Our algorithm is implemented in a freely available R-package, riboVI, which can be found at https://github.com/dcannonwalker/riboVI.

Algorithm 1

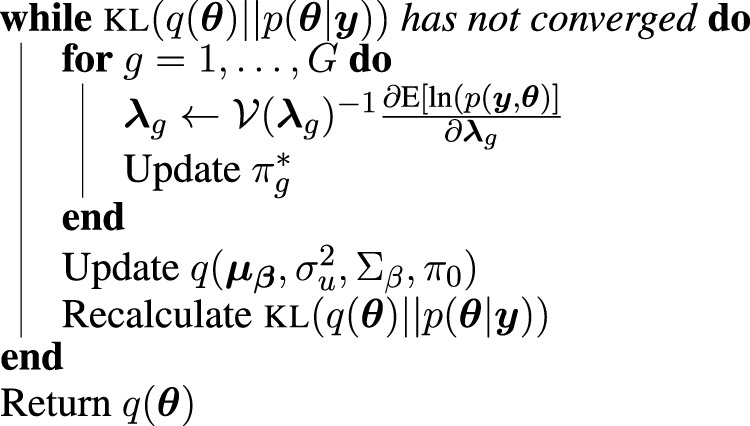

riboVI

### 2.3 Multiple testing

Detecting DTGs amounts to testing for each gene whether *β*_*g*3_ is 0. Since there will be thousands of genes to test in most experiments, multiple test correction is an important consideration in the statistical analysis of Ribo-seq data.

For our model, we can calculate the Bayesian false discovery rate (FDR) using posterior probabilities that each gene is not differentially translated. If we have a collection of *G* posterior probabilities *p*_1_, *…* , *p*_*G*_, representing the posterior probability that each gene is not differentially translated, then we can calculate Bayesian FDR if rejecting $H_{0}^{g}$ when *p*_*g*_ < *c*, by

$$\hat{FDR}\left(c\right)=\frac{\sum_{g}p_{g}\text{I}\left(p_{g}<c\right)}{\sum_{g}\text{I}\left(p_{g}<c\right)}.$$ (12)

Then Bayesian FDR can be controlled at level *α* by finding

$$c^{*}=\sup\left\{c:\hat{FDR}\left(c\right)<\alpha \right\}$$ (13)

### 2.4 Collection of real data

Ribo-seq was performed essentially as described in (Hsu et al., 2016; Chung et al., 2020; Kanodia et al., 2020). Details of methods and experimental rationale may be found in Kanodia (2021), and a more detailed interpretation of results will be described in a forthcoming publication. *Arabidopsis* plants (Col-0, dcl2-1/dcl4-2t) were rub inoculated with sap from uninfected (mock) or RCNMV-infected Nicotiana benthamiana plants (Kanodia and Miller, 2022). Ribosome-protected fragments (RPFs) were isolated from young, uninoculated tissue from infected plants and mock-infected plants at 5 and 8 days post inoculation (DPI). 5 DPI was the earliest time point at which viral RNA was detectable in the uninoculated tissue, and by 8 DPI, RNA levels were nearing their peak. For each time point, each sample of plant tissue (5 biological replicates per time point in the infected condition and 4 replicates per time point in the mock condition) was divided into two halves for analysis. One half was subjected to RPF isolation, the other was subjected to RNA-seq analysis of total RNA. Both the RPF pool and the total RNA pool were sequenced on an Illumina NovaSeq 6000. The quality of raw sequencing reads was assessed using FastQC v.0.11.7 (Andrews et al., 2010) and adapters were removed using Cutadapt v.2.5 (Martin, 2011). RiboToolkit (Liu et al., 2020) was used to determine the RPF lengths with high triplet periodicity and assess the frame enrichment, which was very high for 28 nt fragments. Noncoding RNA sequences were identified for removal using Bowtie v.1.2 (Langmead et al., 2009). The ncRNA-unaligned reads were then mapped to *Arabidopsis* reference genome (TAIR10) using STAR v.2.5 (Dobin et al., 2013) to yield uniquely-mapped reads. The vast majority of RPFs were 28 nt in length, consistent with other reports for *Arabidopsis* (Hsu et al., 2016; Chung et al., 2020), and mapped to coding regions.

## 3 Results

### 3.1 Simulation studies

In order to evaluate the utility of our proposed method, we compared the performance of our method riboVI with the six methods described in the introduction (edgeR, DESeq2, baySeq, xtail, babel, and RiboDiff) using four sets of simulation studies. Simulations A, B, and C are model-based simulations representing progressively larger deviations from our assumed model. Simulation D is a real data-based simulation where the actual distribution of data is unknown.

Model parameters for simulation studies A and B are based on estimates from the real dataset described in Section 2.4. Given these real-data-based estimates as parameters, counts in these studies are entirely simulated. We explain the simulation procedure briefly. We first used edgeR to fit a generalized linear model with the mean (*λ*_*gij*_) modeled as in Eq. 1 and obtained estimates for the fixed effects ***β***_*g*_ and the sample effects *u*_*gi*_ for each gene (edgeR assumes both are fixed). Then we used the empirical means and variances of the estimated *β*_*gp*_ (*p* = 0, *…* , 3) as the means and variances of normal distributions from which to draw *β*_*gp*_ parameters. For approximately 80% of genes in each simulated data set, we set the *β*_*g*3_ parameter to 0 so that there was no difference in translational efficiency between treatments for those genes. The remaining 20% of genes retained the non-zero value simulated from the normal distribution for *β*_*g*3_ and were considered to be true positives for the evaluation of method performance. We used the variance of the estimated *u*_*gi*_ as the variance of a normal distribution centered at 0 from which to draw *u*_*gi*_ (*i* = 1, *…* , 2*n*). After ***β***_*g*_ and the *u*_*gi*_ were drawn, we calculated the mean for each combination of sample and preparation (RPF or RNA-seq) according to Model 1) for each of *G* = 10,000 genes. Based on these means, count data were simulated from Poisson distributions in Simulation A and from negative binomial distributions in Simulation B. The dispersion parameters for the negative binomial distributions in Simulation B were drawn from the empirical distribution of the gene-specific dispersion values that **edgeR** estimated for the same real dataset. For both simulation studies, we have two settings with sample sizes *n* = 2 and *n* = 4.

Simulation C is inspired by the simulation studies described in the supplemental materials of the RiboDiff paper (Zhong et al., 2017). First, we simulated separate means for RPF and mRNA for each of *G* = 10,000 genes from a negative binomial distribution. We then simulated fold changes from a gamma distribution with parameter values estimated from a real data set, as given in Zhong et al. (2017). For three randomly selected subsets of genes, we used the simulated fold changes to modify the means for either mRNA, RPF, or both, respectively. As in simulations A and B, approximately 80% of genes in each simulated data set had no difference in expected translational efficiency between conditions. Finally, we modified the means for paired mRNA and RPF samples with a shared random effect simulated from a Normal distribution. Count data were then generated from a negative binomial distribution with dispersion parameter as a function of the mean, as specified in (Zhong et al., 2017), for each gene. We have two settings with *n* = 2 or *n* = 4 per condition.

Simulation D is based on resampling from the real data set described in Section 2.4. The four samples in the mock condition of the real data set described in Section 2.4 were used to construct simulated data sets. In order to produce multiple unique simulated data sets, for each simulated data set we randomly selected two samples (n = 2) to form a simulated “treatment” condition and the other two samples (n = 2) to form a simulated “control” condition. Then, we simulated fold changes from a shifted gamma distribution similar to that specified in Xiao et al. (2016) with shape parameter 0.6, scale parameter 0.5, and positive shift of 1.5, which we used to modify the counts for mRNA, RPF, or both for three randomly selected subsets of genes, respectively. As in simulations A, B, and C, approximately 80% of genes in each simulated data set had no difference in expected translational efficiency between conditions. Genes with differential translational efficiency (simulated true positives) had a log-scale change in expected translational efficiency of at least 0.4 as a consequence of the positive shift applied to the gamma distribution, representing a further departure from our model’s assumptions. We replicated this procedure to produce each simulated data set that included *G* = 10,000 genes each.

**FIGURE 1:**
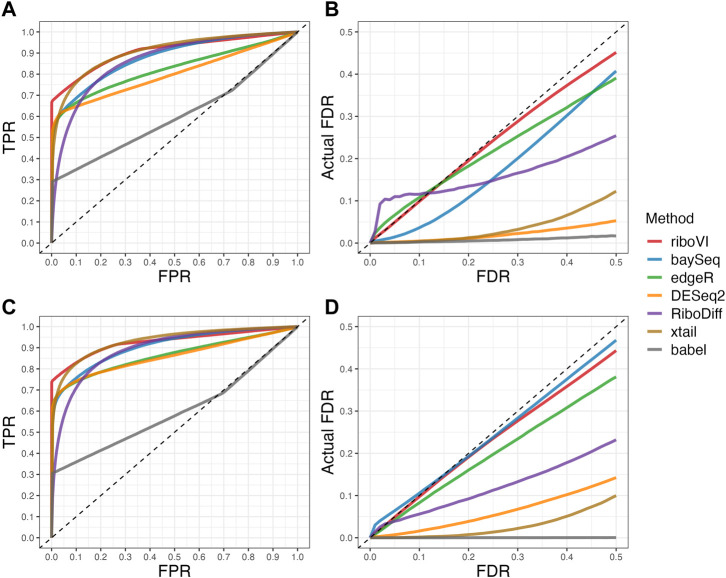
Results for simulation study A **(A)** ROC curves for each method with two replicates per condition; **(B)** FDR curves for each method with two replicates per condition; **(C)** ROC curves for the setting with four replicates per condition. **(D)** FDR curves for the setting with four replicates per condition. Note that both ROC curves and FDR curves are averaged over 100 simulated datasets for each setting.

**FIGURE 2:**
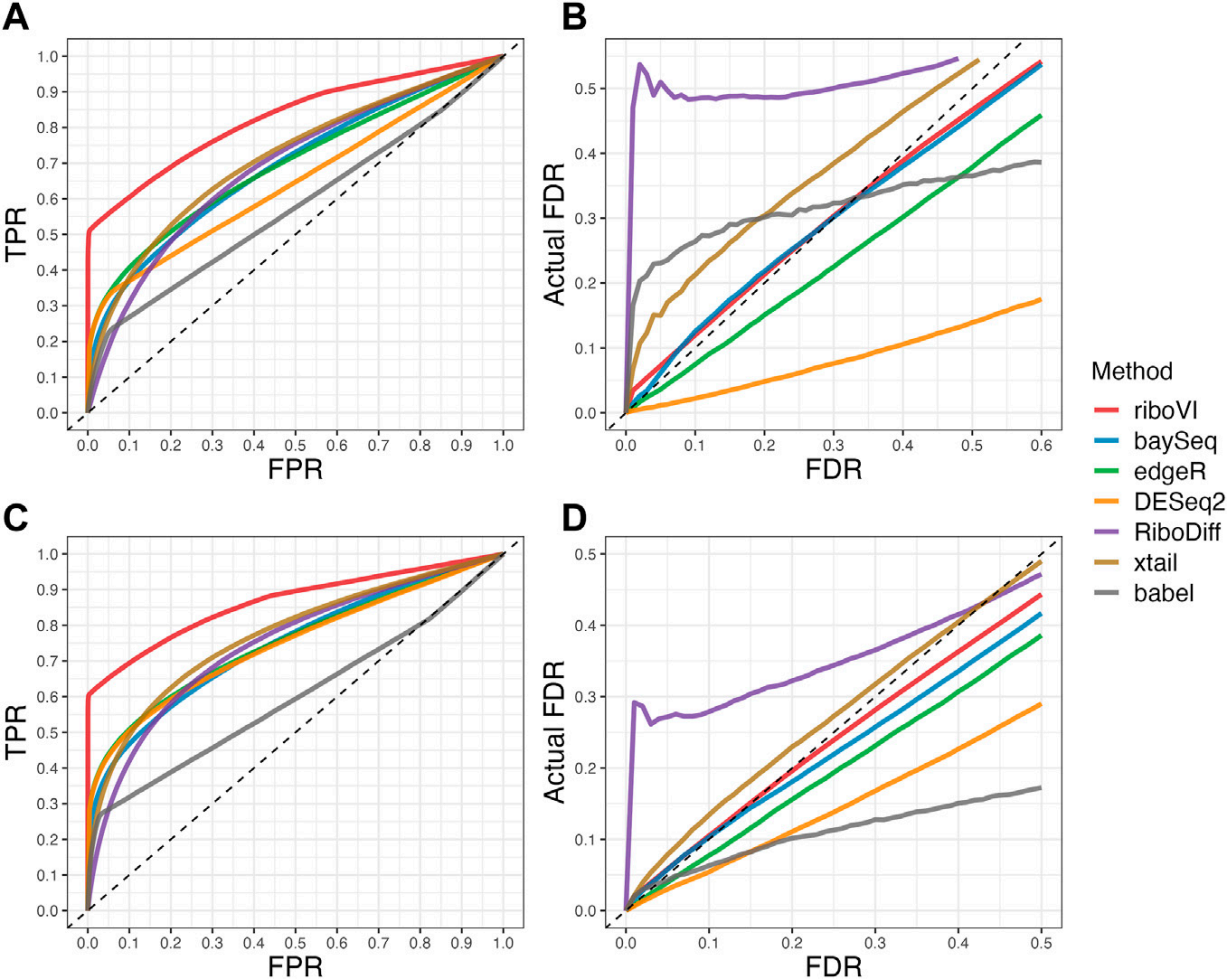
Results for simulation study B **(A)** ROC curves for the setting with two replicates per condition; **(B)** FDR curves for the setting with two replicates per condition; **(C)** ROC curves for the setting with four replicates per condition; **(D)** FDR curves for the setting with four replicates per condition. Note that both ROC curves and FDR curves are averaged over 100 simulated datasets for each setting.

**FIGURE 3:**
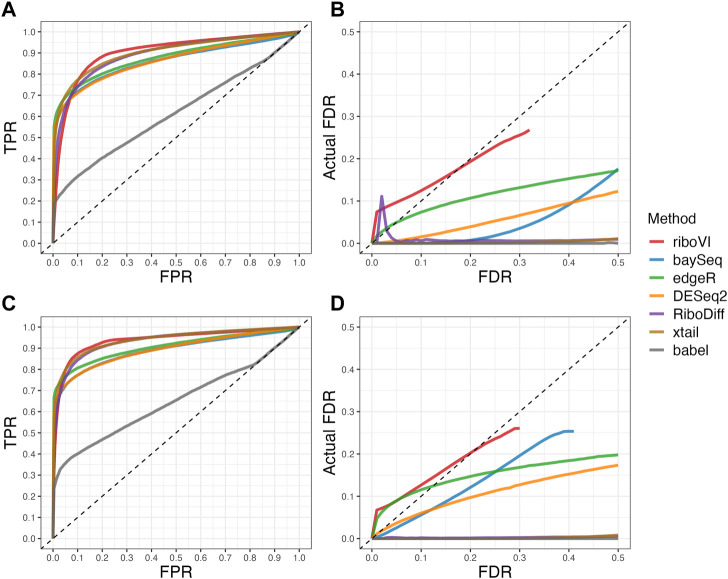
Results for simulation study C **(A)** ROC curves for the setting with two replicates per condition; **(B)** FDR curves for the setting with two replicates per condition; **(C)** ROC curves for the setting with four replicates per condition; **(D)** FDR curves for the setting with four replicates per condition. Note that both ROC curves and FDR curves are averaged over 100 simulated datasets for each setting.

**FIGURE 4:**
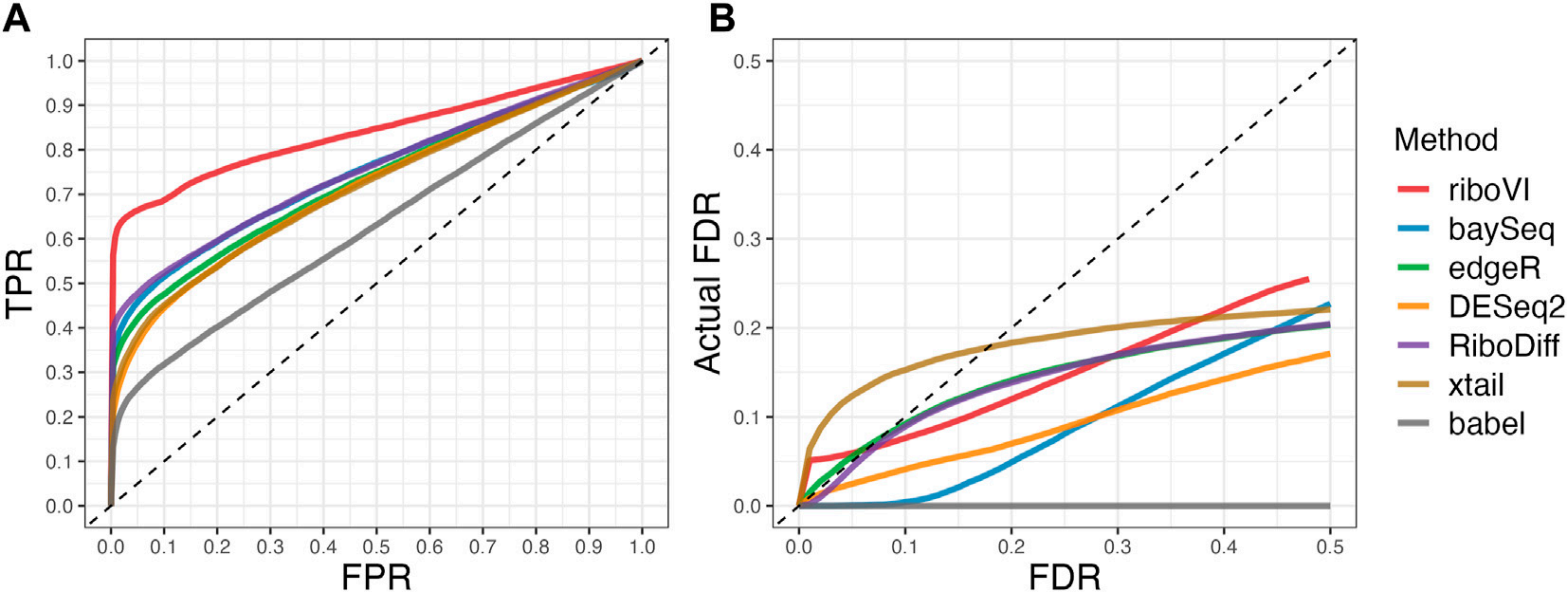
Results for simulation study D **(A)** ROC curves; **(B)** FDR curves. This real-data based simulation includes two replicates per condition, and both ROC curves and FDR curves are averaged over 50 simulated datasets.

We evaluated each method’s ability to classify DTGs using ROC curves (receiver operating characteristic curves) by plotting the true positive rate (TPR) against the false positive rate (FPR). The ROC curves were averaged over 100 datasets for each simulation setting and presented in Figures 1–4 for simulations A–D, respectively. The area under the curves (AUCs) are shown in Table 3. RiboVI and xtail have similar detection accuracy for Simulation A, and they outperform the other methods (Figure 1; Table 3). BaySeq, another method that models the paired structure, also performs relatively well in Simulation A, especially when sample size is larger. RiboVI dramatically outperforms all the other methods in classifying DTG genes in Simulation B (Figure 2; Table 3). This suggests that although our assumed model is not exactly negative binomial (based on which data were generated for Simulation B), RiboVI still provides outstanding performance possibly due to its ability to handle overdispersion by including the random sample effect. The methods based on negative binomial models with fixed sample effect (edgeR, and DESeq2) do not show advantages in Simulation B, possibly due to the challenges of estimating several mean parameters and a dispersion parameter for each gene with a small number of replicates. RiboVI, xtail, and RiboDiff have similar detection accuracy for Simulation C, again outperforming the other methods (Figure 3; Table 3). RiboVI dramatically outperforms all the other methods in Simulation D where data were simulated based on a real dataset (Figure 4; Table 3) and most closely mimic real applications. Overall, the proposed method (riboVI) exhibits detection accuracy superior or equivalent to the besting performing method among those under comparison across all simulation studies.

**TABLE 3:** Mean area under the ROC curve (AUC) for each method and simulation study, with either n = 2 or n = 4 replicates per condition. Standard deviation is included in the parenthesis next to the corresponding mean.

		Simulation study A		Simulation study B		Simulation study C		Simulation study D
	method	n = 2	n = 4	n = 2	n = 4	n = 2	n = 4	n = 2
1	babel	0.6 (0.007)	0.6 (0.006)	0.576 (0.008)	0.598 (0.007)	0.615 (0.077)	0.651 (0.09)	0.628 (0.024)
2	baySeq	0.88 (0.007)	0.907 (0.008)	0.692 (0.008)	0.746 (0.007)	0.861 (0.026)	0.891 (0.031)	0.751 (0.016)
3	DESeq2	0.8 (0.007)	0.86 (0.007)	0.646 (0.01)	0.748 (0.007)	0.864 (0.032)	0.895 (0.035)	0.718 (0.021)
4	edgeR	0.824 (0.018)	0.865 (0.015)	0.696 (0.007)	0.752 (0.007)	0.878 (0.035)	0.907 (0.036)	0.732 (0.035)
5	riboVI	0.908 (0.005)	0.928 (0.004)	0.827 (0.007)	0.864 (0.006)	0.903 (0.014)	0.939 (0.01)	0.841 (0.007)
6	RiboDiff	0.866 (0.005)	0.894 (0.005)	0.691 (0.007)	0.747 (0.007)	0.894 (0.019)	0.935 (0.024)	0.755 (0.013)
7	xtail	0.906 (0.004)	0.933 (0.004)	0.713 (0.008)	0.771 (0.007)	0.904 (0.034)	0.941 (0.03)	0.718 (0.021)

We also evaluated each method’s ability to control multiple testing errors by comparing the actual FDR to nominal levels (Figures 1–4 for Simulations A–D). Tables of actual FDRs at nominal levels 0.05 and 0.1 are provided in the supplementary materials. RiboVI, baySeq, DESeq2 and edgeR typically control FDR to nominal levels or below, although DESeq2 and baySeq can be very conservative in some settings. RiboDiff, babel, and Xtail do not control FDR well in general, and their performance is inconsistent across simulation studies: they can be quite conservative in some cases but liberal in others. RiboVI performs best in simulations A and B (see Figures 1, 2), where the actual FDR are very close to the nominal levels. It does not perform as well in simulations C (Figure 3) and D (4) but its performance is still acceptable and better than other methods. Overall, RiboVI controls FDR close to the nominal levels and is the best method among all being compared.

### 3.2 Real data analysis

In addition to our simulation studies, we used riboVI, edgeR, and xtail to analyze a data set from a real Ribo-seq study on Arabidopsis plants, investigating the effect of infection by red clover necrotic mosaic virus (RCNMV). We collected leaves from the mock-group and infected plants at 5 and 8 days post inoculation (DPI) and performed Ribo-seq experiments as described in Section 2.4. We selected edgeR and xtail because they are the methods that are most competitive with riboVI across simulations studies and because they produce quantitative estimates of translational efficiency change. These methods are also relatively accessible and user-friendly for practitioners. Since edgeR and DESeq2 are based on very similar models and edgeR consistently outperforms DESeq2 in simulations, we did not include DESeq2 in the real data analysis.

**FIGURE 5:**
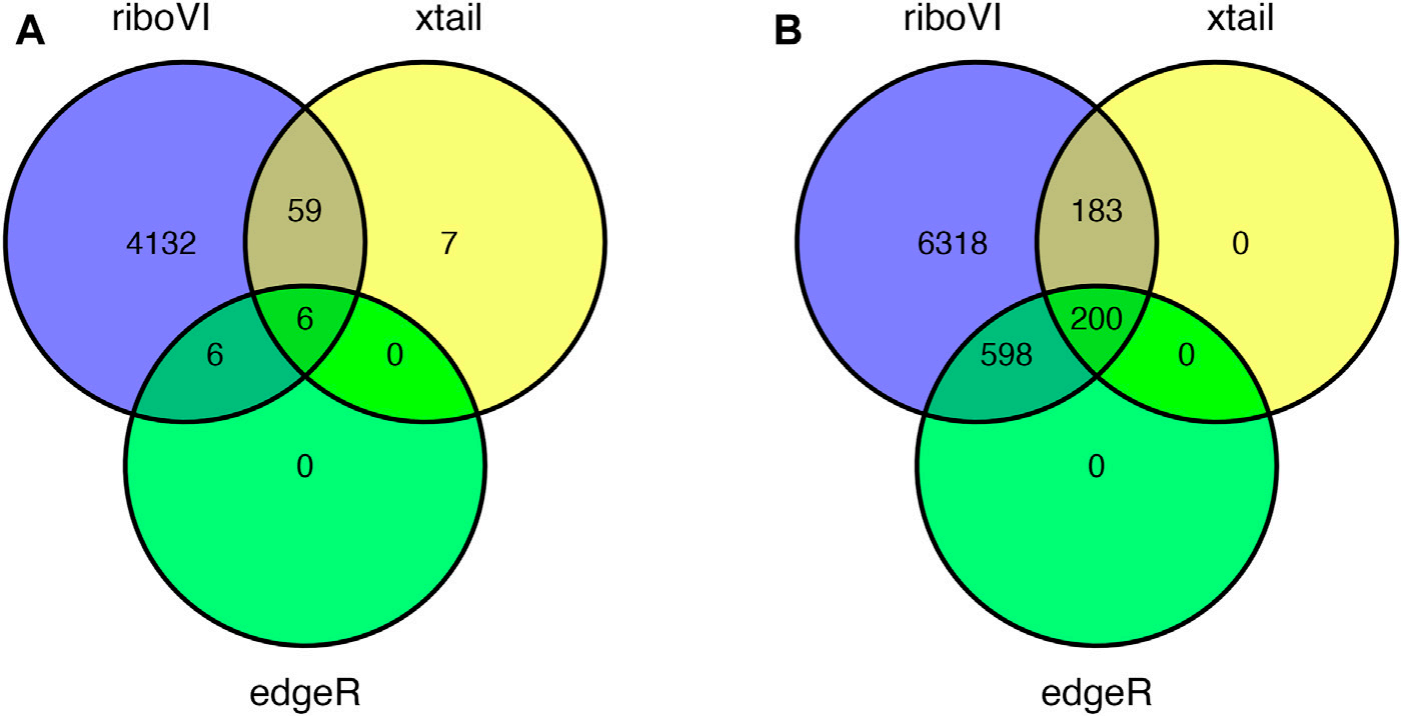
Overlap in DTGs identified by each method at 5 or 8 days post inoculation and FDR = 0.05: **(A)** 5 DPI; **(B)** 8 DPI.

**FIGURE 6:**
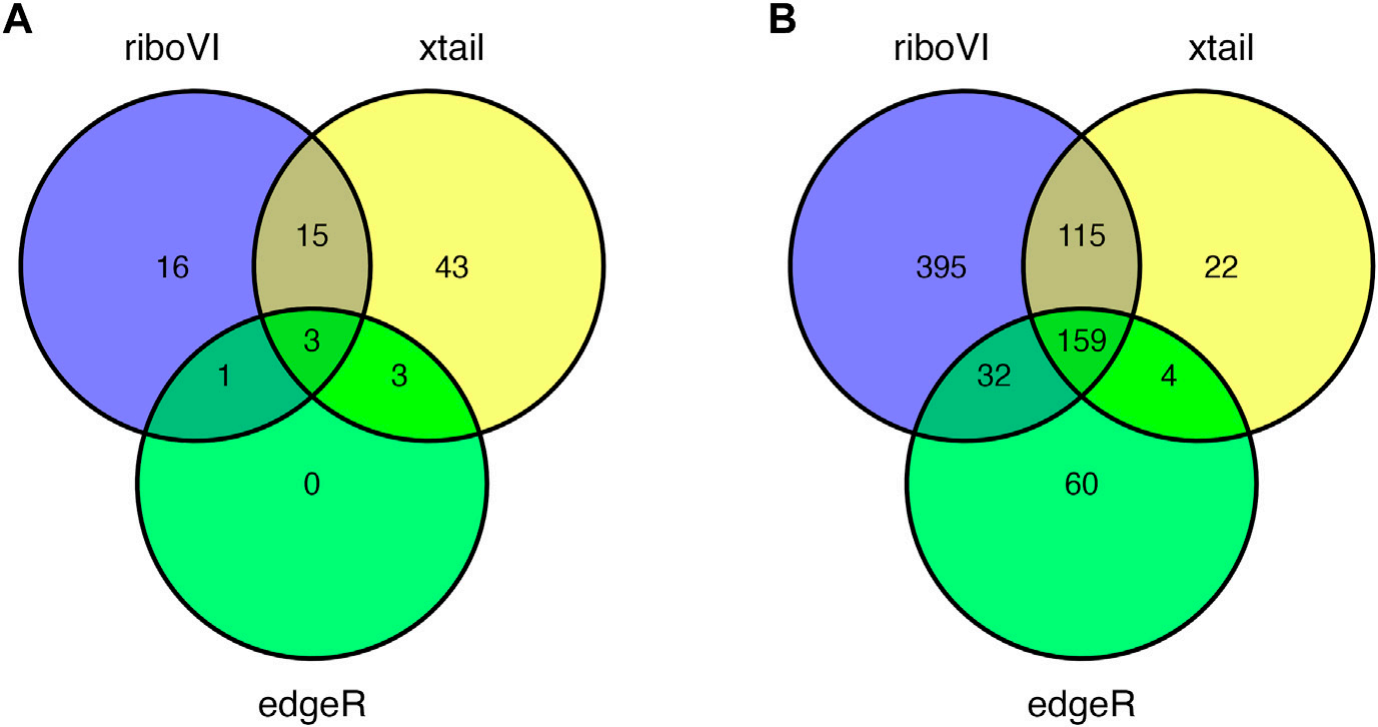
Overlap in DTGs identified by each method with FDR = 0.05 and including only genes with absolute estimated log2 translational efficiency change >0.5 : **(A)** 5 DPI; **(B)** 8 DPI.

Prior to analysis, we filtered out genes with a large proportion of 0 counts across all samples, retaining *G* = 17,388 genes. After analyzing this dataset using riboVI, edgeR, and xtail, we first compared the number of genes classified by each method as exhibiting differential translational efficiency at nominal FDR levels 0.05 and 0.1. RiboVI identified substantially more genes than the other two methods at the same nominal FDR level, as shown in Figure 5. This is consistent with the results from simulation studies, where riboVI demonstrated the best power and FDR control. Nearly all genes identified by edgeR or xtail at a given FDR level were also identified by riboVI. We also considered the absolute value of the estimated translational efficiency change parameter, or *β*_*g*3_ in Model (2.1), as a way to identify genes whose change in translation is more likely to be associated with substantial effects on the biological system. Hence, we retained only the genes with an estimated translational efficiency change with an absolute value greater than 0.5 (in log2 scale) in addition to the FDR threshold for the functional analysis next. Overlap in genes detected by the three methods using this additional criterion is shown in Figure 6.

To investigate the functions of genes identified by each method, we performed GO enrichment analyses. GO analysis was conducted with the Panther Gene List Analysis webserver (http://www.pantherdb.org/) (Mi et al., 2019; Thomas et al., 2022) where gene IDs, grouped by their expression level and time point (5 dpi and TE up, 5 dpi and TE down, etc.), were tested for statistical overrepresentation as compared to the default gene set for *Arabidopsis thaliana* maintained by PantherDB and in the context of biological processes. Revigo tree maps from the GO analysis are presented in supplementary figures. RiboVI identified more genes exhibiting differential translational efficiency, which enabled the detection of more specific GO terms as being over-represented than the other methods. For example, at 5 DPI riboVI was the only method which identified enough genes as being significantly up-translated to return significantly over-represented GO terms. For genes that showed a decrease in translational efficiency at 5 DPI, both riboVI and xtail identified enough for GO analysis, but all 22 GO terms found by xtail were also found by riboVI, while riboVI found an additional 222 overrepresented GO terms that were not identified by xtail.

The larger number of overrepresented GO terms found by riboVI facilitates the interpretation of results, in part because the increased specificity of GO terms from riboVI helps provide a detailed explanation of the biological system under study. For example, GO analysis of xtail-identified genes revealed an overrepresentation of signal transduction (GO:0007165), when analysis of riboVI-identified genes revealed not only signal transduction (GO:0007165) but also hormone-mediated signaling pathway (GO:0009755) and regulation of signal transduction (GO:0009966) thereby providing a more detailed understanding of the system. GO analysis of riboVI identified genes at 5 DPI also revealed terms not identified at all by analysis of xtail identified genes. Specific examples include terms such as cytoplasmic translational initiation (GO:0002183) and rRNA processing (GO:0006364). These findings, unique to riboVI, make biological sense considering single-stranded positive sense RNA viruses first co-opt host translation factors upon infection to produce viral proteins for replication. The results for genes identified at 8 DPI were similar in the sense that riboVI returned more numerous and specific terms which are biologically meaningful. More specific biological insights will be presented in a separate paper.

## 4 Discussion

In this paper we propose a Bayesian hierarchical model for Ribo-seq data anaysis that incorporates a random sample effect to accommodate the experimental design factors. We derive a computational algorithm to fit our model and compare the performance of our method to the other methods available for analyzing Ribo-seq. Based on a variety of simulation studies where data were *not* generated based on our model, our method riboVI outperforms the other methods with higher power for detecting genes that exhibit differential translational efficiency and better control of FDR.

In an analysis of real data, riboVI identifies more genes as exhibiting differential translational efficiency than xtail or edgeR at the same nominal FDR. This is not surprising based on simulation results that show riboVI has better power and controls FDR to the desired level. The biological inferences for the detected DTGs need to be validated using complementary evidence. In this paper, we apply functional analysis of the detected DTGs through GO enrichment analyses, which show that riboVI identifies a larger number of more specific GO terms than the other two methods. The additional GO terms that riboVI identifies are associated with functions likely to be affected by viral infection, which makes biological sense. The increased information derived from the GO enrichment analysis of the riboVI gene set facilitates the interpretation of results and helps provide a more detailed understanding of the biological system. In future research, benchmark Ribo-seq data sets may be generated and further validate our method’s performance.

A barrier to applying current Ribo-seq analysis methods such as xtail and RiboDiff is their inability to represent experimental designs with more than a single treatment with two conditions. As the complexity of Ribo-seq experiments grows, these methods will not be able to perform the requisite analysis. Our method riboVI is based on a GLMM framework and can easily incorporate different design structures. Note that our current code implementation is limited to 2-treatment comparisons, and we are developing this extension to our codebase, which we hope to release in a follow-up version of the riboVI package.

While riboVI has higher power than other methods for detecting DTGs, we notice that its estimated translational efficiency changes tend to be smaller than other methods. This is likely due to the shrinkage effect on the parameter *β*_*g*3_ from our model. This effect is more pronounced when only a small proportion of genes in a data set exhibit substantial changes in translational efficiency. One area for future research is to improve the accuracy of *β*_*g*3_ estimates, and this might be done by modifying the mixture prior for *β*_*g*3_ in Model 2.1. Also, with Bayesian posterior inference, we can modify the hypothesis to test and aim to only identify those genes with a large enough translational efficiency change.

In summary, our method provides a useful advancement over available methods for differential translational efficiency analysis of Ribo-seq data. It demonstrates superior power and FDR control to detect DTGs in comparison with other methods in both simulation studies and in applications to real data. We provide an R package called riboVI which is freely available at https://github.com/dcannonwalker/riboVI.

## Data Availability

The original Ribo-Seq data presented in the study are publicly available. This data can be found here: NCBI BioProject accession number PRJNA950066, https://dataview.ncbi.nlm.nih.gov/object/PRJNA950066.
