# Supplementary material for: Variational inference for detecting differential translation in ribosome profiling studies
Source: Front Genet. 2023 Jun 23;14:1178508. doi: 10.3389/fgene.2023.1178508 (PMC10326721; doi:10.3389/fgene.2023.1178508)
Supplement: Supplementary file 1 [file DataSheet1.PDF]

Supplementary Material

1 Variational updates 1
  1.1 Conjugate updates 1
  1.2 Non-conjugate updates 3
2 Distribution of Random Effects 4
  2.1 Point estimates from real data 4
  2.2 Simulation distribution 4
3 Identification of DTGs 4
  3.1 Heatmaps for Real Data 4
  3.2 Parameter estimates from Simulation D 4
4 True positive and false positive calls at FDR 0.05 4
5 Tree maps from GO analysis 4
6 Supplementary Tables and Figures 5
  6.1 Tables 5
  6.2 Figures 5

1 VARIATIONAL UPDATES

1.1 Conjugate updates

1.1.1 Update for q(**D**)

Below is the derivation for the optimal form of the variational distribution for $\boldsymbol{D}$, $q^*(\boldsymbol{D})$, starting from equation S1, which reproduced from the main text for reference. As in the main text, q^*_m is a generic placeholder for the optimal value of a single component factor of the variational distribution, in this case $q^*(\boldsymbol{D})$.

$$\ln(q^*_m) = \underset{-m}{\mathrm{E}}[\ln(p(\boldsymbol{y}, \boldsymbol{\theta}))] + \text{constant} \tag{S1}$$

$$\begin{aligned}
\ln(q^*(\boldsymbol{D})) &= \underset{-q(D)}{\mathrm{E}}[\ln(p(\boldsymbol{y}, \boldsymbol{\theta}))] + \text{constant} \\
&= \sum_g [D_g \rho_{g1} + (1 - D_g)\rho_{g2}] + \text{constant} \\
\rho_{g1} &= (\mathrm{E}[\ln(p(\boldsymbol{y_g}|\boldsymbol{\beta}^*_g, \boldsymbol{u}_g, D_g = 1))] + \ln(\pi_0)) \\
\rho_{g2} &= \mathrm{E}[\ln(p(\boldsymbol{y_g}|\boldsymbol{\beta}^*_g, \boldsymbol{u}_g, W_g, D_g = 0))] + \ln(1 - \pi_0)
\end{aligned} \tag{S2}$$

So $q^*(\boldsymbol{D})$ factors into $\prod_g q^*(D_g)$, each of which is a Bernoulli distribution with mean parameter π_g^* in S3.

$$\begin{aligned} \pi_g^* &= \frac{r_{g1}}{r_{g2} + r_{g1}} \\ r_{g1} &= \exp(\rho_{g1}) \\ r_{g2} &= \exp(\rho_{g2}) \end{aligned} \tag{S3}$$

Looking at this update more closely:

$$\begin{aligned} \pi_g^* &= (1 + \frac{r_{g2}}{r_{g1}})^{-1} \\ &= (1 + \exp(\rho_{g2} - \rho_{g1}))^{-1} \end{aligned} \tag{S4}$$

This will be close to 0 if the sum of the expected value of the log likelihood and log prior for $D_g = 0$ (corresponding to the alternative hypothesis) is larger than that for $D_g = 1$, under the current variational distribution.

1.1.2 Other conjugate updates

Update $q(\boldsymbol{\mu}_\beta)$

$$\begin{aligned} \ln(q^*(\boldsymbol{\mu}_\beta)) &= \underset{-q(\mu_\beta)}{\mathrm{E}}[\ln(p(\boldsymbol{y}, \boldsymbol{\theta}))] + \text{constant} \\ &= \underset{-q(\mu_\beta)}{\mathrm{E}} \left[\sum_g \ln(p(\boldsymbol{\beta}_g^*, W_g | \boldsymbol{\mu}_\beta, \boldsymbol{\Sigma}_\beta)) + \ln(p(\boldsymbol{\mu}_\beta)) \right] + \text{constant} \\ &= \sum_g \left[\boldsymbol{\mu}_g^T (E\boldsymbol{\Sigma}_\beta^{-1}) \boldsymbol{\mu}_\beta - \frac{1}{2} \boldsymbol{\mu}_\beta^T (E\boldsymbol{\Sigma}_\beta^{-1}) \boldsymbol{\mu}_\beta \right] - \frac{P}{2} (E\sigma_0^{-2}) \boldsymbol{\mu}_\beta^T \boldsymbol{\mu}_\beta + \text{constant} \\ &= -\frac{1}{2} (\boldsymbol{\mu}_\beta - M)^T R^{-1} (\boldsymbol{\mu}_\beta - M) + \text{constant} \\ R &= G(E\boldsymbol{\Sigma}_\beta^{-1}) + (E\sigma_0^{-2}) I_P \\ M &= R[\sum_g (E\boldsymbol{\Sigma}_\beta^{-1}) \boldsymbol{\mu}_g] \end{aligned} \tag{S5}$$

Update $q(\sigma_{\beta p}^{-2})$ (update for $q(\sigma_u^{-2})$ is essentially the same form)

Let $\tau_{\beta p} = \sigma_{\beta p}^{-2}$

$$\begin{aligned}
\ln(q^*(\tau_{\beta p})) &= \underset{-q(\tau_{\beta p})}{\mathrm{E}}[\ln(p(\boldsymbol{y}, \boldsymbol{\theta}))] + \text{constant} \\
&= \underset{-q(\tau_{\beta p})}{\mathrm{E}}\left[\sum_g \ln(p(\boldsymbol{\beta}_g^*, W_g|\boldsymbol{\mu}_\beta, \boldsymbol{\Sigma}_\beta)) + \ln(p(\tau_\beta))\right] + \text{constant} \\
&= -\frac{1}{2}\left[\gamma_p + \sum_g E(\beta_{gp} - \mu_{\beta p})^2\right]\tau_{\beta p} + (G + \alpha_p - 1)\ln\tau_{\beta p} + \text{constant}
\end{aligned} \tag{S6}$$

1.2 Non-conjugate updates

Restating the exponential family assumption from the main text:

$$\begin{aligned}
\boldsymbol{\kappa}_g &:= (\boldsymbol{\beta}_g^*, W_g, \boldsymbol{u}_g)^T \\
q(\boldsymbol{\kappa}_g) &= \exp(\boldsymbol{\lambda}_g^T t(\boldsymbol{\kappa}_g) - h(\boldsymbol{\lambda}_g))
\end{aligned} \tag{S7}$$

$$\boldsymbol{\lambda}_g \leftarrow \mathcal{V}(\boldsymbol{\lambda}_g)^{-1}\frac{\partial \mathrm{E}[\ln(p(\boldsymbol{y}, \boldsymbol{\theta})]}{\partial \boldsymbol{\lambda}_g} \tag{S8}$$

In our case, the exponential-family in S7 will be the multivariate normal distribution; $\boldsymbol{\lambda}_g$ and $t(\boldsymbol{\kappa}_g)$ will be the natural parameter and sufficient statistic of the MVN.

For the case where $q(\boldsymbol{\kappa}_g)$ is the MVN distribution, Wand (2014) offers a simplified update in terms of the mean and variance parameters. This is reproduced in equation S9, where 'vec' denotes the operation defined in Magnus and Neudecker (2019) that maps a matrix to a vector, and $\mathrm{D}_x s$ denotes the vector of derivatives of s w.r.t. x.

Update $q_g(\boldsymbol{\beta}_g^*, W_g, \boldsymbol{u}_g)$

$$\begin{aligned}
\boldsymbol{\Sigma}_g &\leftarrow (-2\ \mathrm{vec}^{-1}\mathrm{D}_{\mathrm{vec}(\Sigma_g)}\mathrm{E}[\ln p(\boldsymbol{y}, \boldsymbol{\theta})])^{-1} \\
\boldsymbol{\mu}_g &\leftarrow \boldsymbol{\mu}_g + \boldsymbol{\Sigma}_g^{-1}\mathrm{D}_{\mu_g}\mathrm{E}[\ln p(\boldsymbol{y}, \boldsymbol{\theta})]
\end{aligned} \tag{S9}$$

If $s = \mathrm{E}[\ln p(\boldsymbol{y}, \boldsymbol{\theta})]$, then:

$$\begin{aligned}
\mathrm{vec}^{-1}(\mathrm{D}_{\mathrm{vec}(\Sigma_g)}s) &= -\frac{1}{2}[(E\boldsymbol{\Sigma}_\beta^{-1}) + (1-\pi_g)C^T\mathrm{diag}(\exp[A_1])C + \pi_g C_0^T\mathrm{diag}(\exp[A_0])C_0] \\
\mathrm{D}_{\mu_g}s &= (E\boldsymbol{\Sigma}_\beta^{-1})(M - \boldsymbol{\mu}_g) + (1-\pi_g) * C^T(y_g - \exp[A_1]) + \pi_g C_0^T(y_g - \exp[A_0]) \\
A_1 &= C\boldsymbol{\mu}_g + \frac{1}{2}\mathrm{diag}(C\boldsymbol{\Sigma}_g C^T) \\
A_0 &= C_0\boldsymbol{\mu}_g + \frac{1}{2}\mathrm{diag}(C_0\boldsymbol{\Sigma}_g C_0^T)
\end{aligned} \tag{S10}$$

Where $C = [X, Z]$ and C_0 is C with the column corresponding to β_{g3} set to 0.

2 DISTRIBUTION OF RANDOM EFFECTS

An important innovation of our model is the inclusion of random effects, the u_{gi}, to represent sample-specific effects shared by total mRNA and RPF from the same biological sample. Here we provide additional information about the distribution of these effects estimated from the real data, and the distributions from which we simulate these effects for simulations A, B, and C.

2.1 Point estimates from real data

`RiboVI` estimates one random effect for each gene and sample in the real data. The per-sample distributions of the point estimates for these parameters are shown for the two different time points in the real data in Figures S1 and S2. Simulation D does not introduce any simulated random effects beyond those that are present in the real data, and the distribution of estimated random effects for data sets in this simulation study is essentially identical to that of the real data, see Figure S3.

2.2 Simulation distribution

For simulations A, B, and C, random effects for each gene and sample were simulated from Normal distributions. The variance of the Normal distribution was selected by taking the empirical variance of the set of point estimates for sample effects in our real data set produced by `edgeR`. A comparison of the distribution of `edgeR` estimates and the resulting Normal distribution from which we simulated random effect parameters for simulations A, B, and C is show in Figure S4.

3 IDENTIFICATION OF DTGS

3.1 Heatmaps for Real Data

Figures S5 through S10 show heatmaps of estimated translational stimulation and repression for each method, for the genes included in the Venn-diagram in Figure 5 in the manuscript. Positive values (yellower colors) correspond to translational stimulation; negative values (bluer colors) correspond to translational repression.

3.2 Parameter estimates from Simulation D

Figures S11 and S11 show estimated change in translational efficiency plotted against true (simulated) values for simulation D. Figure S11 shows only false positives and true positives at nominal FDR 0.05; Figure S12 plots estimates for all genes.

4 TRUE POSITIVE AND FALSE POSITIVE CALLS AT FDR 0.05

Table S3 show total true positive and false positive calls at nominal FDR 0.05 for each method for example data sets from each simulation setting. Tables with gene-by-gene counts alongside true and false positive calls for each method are attached in the additional supplementary data file.

5 TREE MAPS FROM GO ANALYSIS

Figures S13 and S14 show Revigo tree maps of GO terms identified by `riboVI` (S13) and `xtail` (S14), as discussed in Section 3.2 of the main text.

REFERENCES

Magnus, J. R. and Neudecker, H. (2019). *Matrix differential calculus with applications in statistics and econometrics* (John Wiley & Sons)

Wand, M. P. (2014). Fully simplified multivariate normal updates in non-conjugate variational message passing. *Journal of Machine Learning Research*

6 SUPPLEMENTARY TABLES AND FIGURES

6.1 Tables

		Simulation study A		Simulation study B		Simulation study C		Simulation study D
	method	n = 2	n = 4	n = 2	n = 4	n = 2	n = 4	n = 2
1	babel	0.001 (0.002)	0 (0)	0.228 (0.076)	0.043 (0.018)	0 (0)	0 (0)	0 (0)
2	baySeq	0.014 (0.015)	0.064 (0.038)	0.062 (0.025)	0.058 (0.015)	0 (0)	0.025 (0.015)	0.001 (0.001)
3	DESeq2	0.002 (0.013)	0.007 (0.017)	0.012 (0.007)	0.03 (0.008)	0.006 (0.005)	0.037 (0.025)	0.025 (0.013)
4	edgeR	0.066 (0.079)	0.041 (0.037)	0.036 (0.014)	0.039 (0.01)	0.049 (0.025)	0.09 (0.039)	0.055 (0.04)
5	riboVI	0.049 (0.003)	0.049 (0.003)	0.073 (0.007)	0.058 (0.004)	0.097 (0.014)	0.089 (0.018)	0.059 (0.006)
6	RiboDiff	0.11 (0.063)	0.039 (0.017)	0.51 (0.121)	0.272 (0.061)	0.01 (0.051)	0.002 (0.011)	0.043 (0.017)
7	xtail	0.002 (0.004)	0 (0.001)	0.15 (0.068)	0.079 (0.023)	0 (0)	0 (0.001)	0.124 (0.02)

Table S1. Mean (SD) of proportion of false discoveries among flagged genes (actual FDR) at nominal FDR = 0.05

		Simulation study A		Simulation study B		Simulation study C		Simulation study D
	method	n = 2	n = 4	n = 2	n = 4	n = 2	n = 4	n = 2
1	babel	0.002 (0.003)	0 (0)	0.261 (0.062)	0.064 (0.021)	0 (0)	0 (0)	0 (0)
2	baySeq	0.036 (0.022)	0.106 (0.041)	0.125 (0.027)	0.103 (0.016)	0 (0.001)	0.056 (0.021)	0.004 (0.004)
3	DESeq2	0.005 (0.019)	0.016 (0.023)	0.022 (0.009)	0.054 (0.009)	0.015 (0.01)	0.06 (0.031)	0.041 (0.016)
4	edgeR	0.108 (0.101)	0.083 (0.064)	0.075 (0.021)	0.077 (0.013)	0.074 (0.028)	0.115 (0.039)	0.092 (0.043)
5	riboVI	0.099 (0.004)	0.097 (0.003)	0.12 (0.007)	0.105 (0.005)	0.125 (0.017)	0.127 (0.016)	0.076 (0.007)
6	RiboDiff	0.116 (0.036)	0.056 (0.015)	0.484 (0.078)	0.279 (0.042)	0.008 (0.027)	0.002 (0.005)	0.089 (0.016)
7	xtail	0.004 (0.004)	0.002 (0.002)	0.213 (0.045)	0.134 (0.02)	0 (0.001)	0 (0.001)	0.153 (0.017)

Table S2. Mean (SD) of proportion of false discoveries among flagged genes (actual FDR) at nominal FDR = 0.1

		Simulation study A		Simulation study B		Simulation study C		Simulation study D	
	method	false positive	true positive	false positive	true positive	false positive	true positive	false positive	true positive
1	babel	0	174	6	30	0	0	0	0
2	bayseq	3	769	9	82	0	357	0	281
3	deseq	0	911	0	211	5	782	18	443
4	edger	21	1083	9	273	31	1132	71	710
5	ribodiff	4	32	7	11	0	3	31	753
6	ribovi	75	1355	83	10	167	137	67	1097
7	xtail	1	116	4	31	0	69	157	875

Table S3. True positive and false positive calls for each method at nominal FDR = 0.05 for an example data set from each simulation study, N = 8. Gene-by-gene simulated counts are in the supplementary data file.

6.2 Figures

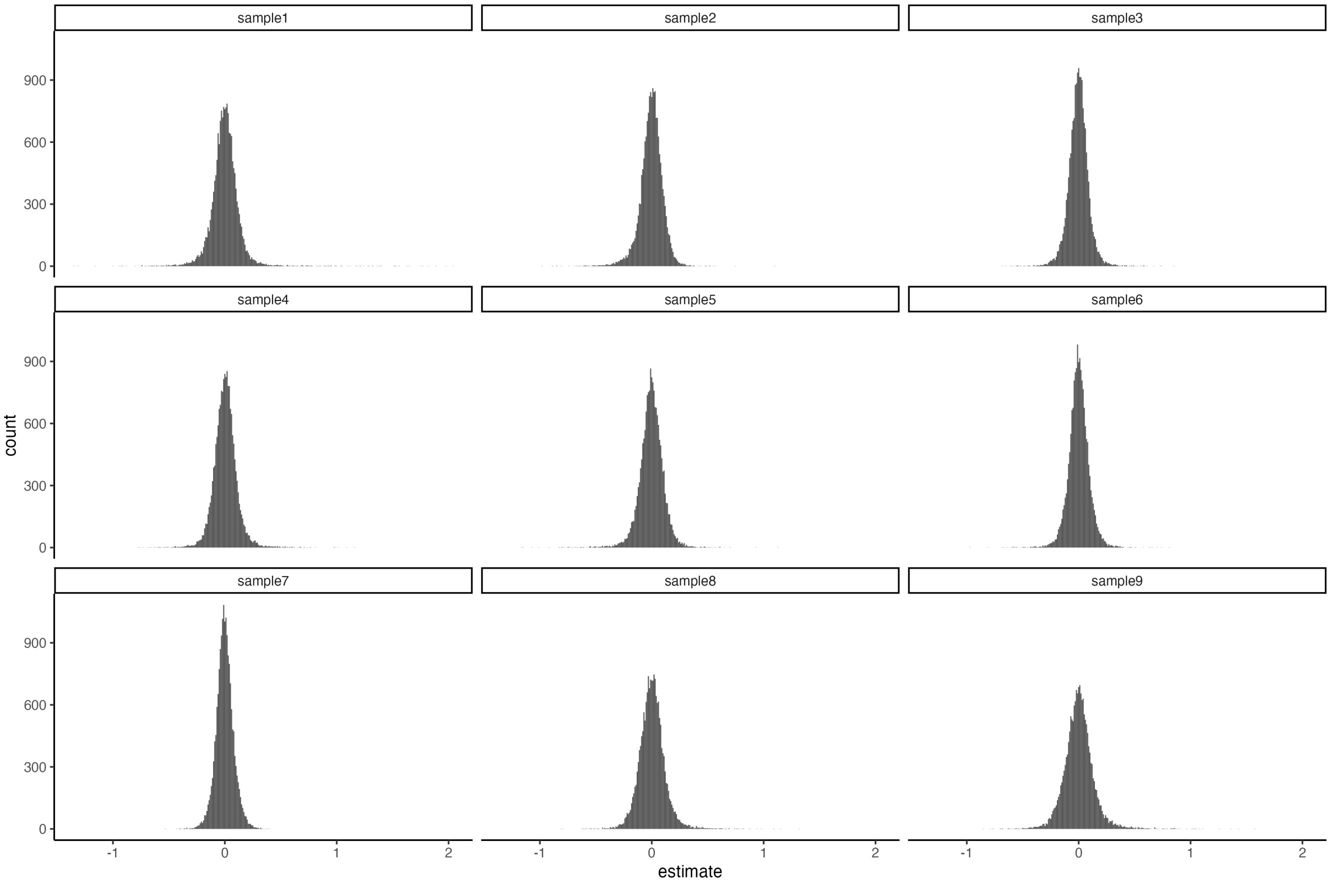

Figure S1. Per-sample distribution of `riboVI` estimated random effects at 5 DPI

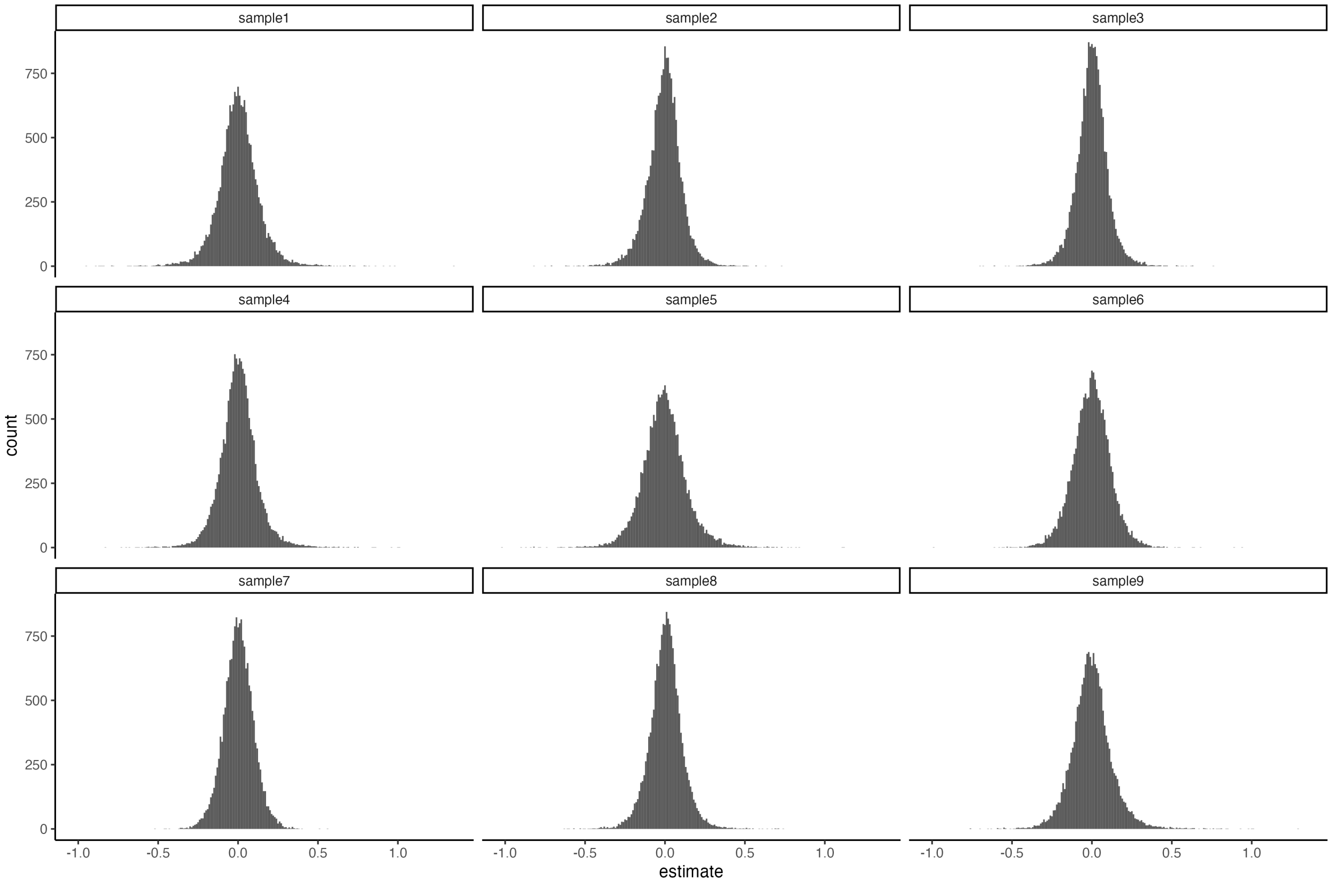

Figure S2. Per-sample distribution of `riboVI` estimated random effects at 8 DPI

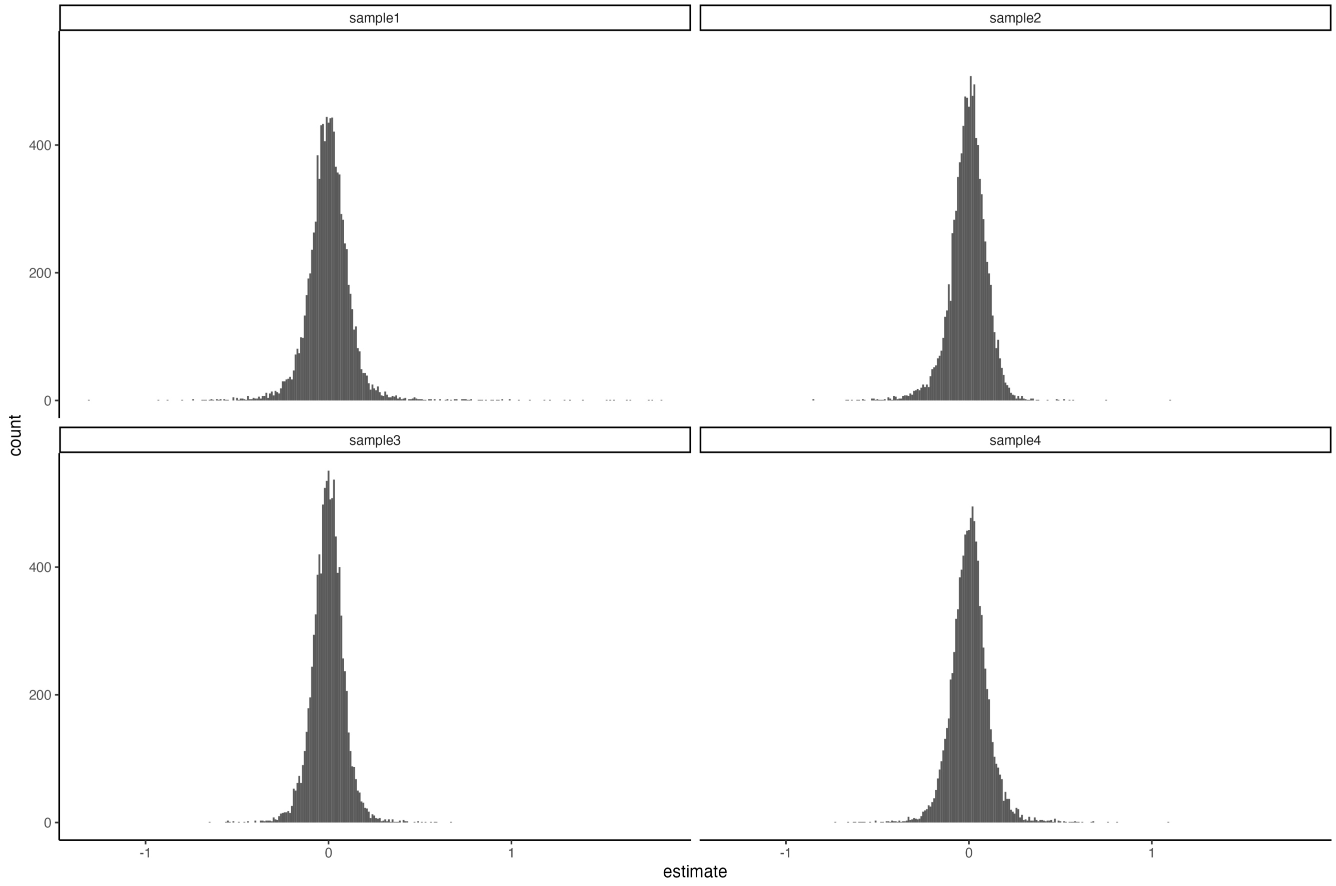

Figure S3. Per-sample distribution of `riboVI` estimated random effects for a representative example data set from simulation setting D, which uses counts from 4 mock-condition samples at 5 DPI

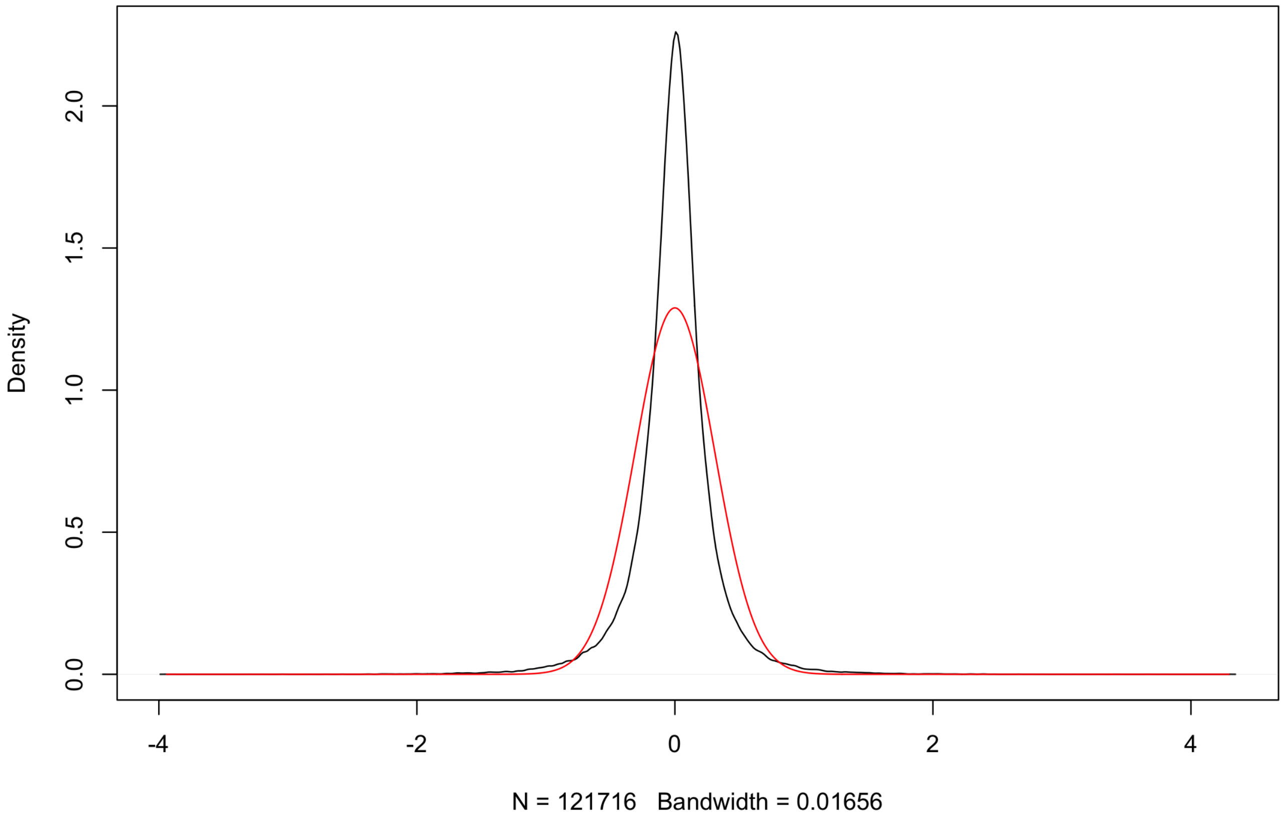

Figure S4. Empirical distribution of sample-specific effect estimates from `edgeR` (black) and the Normal approximation with a matching variance (red)

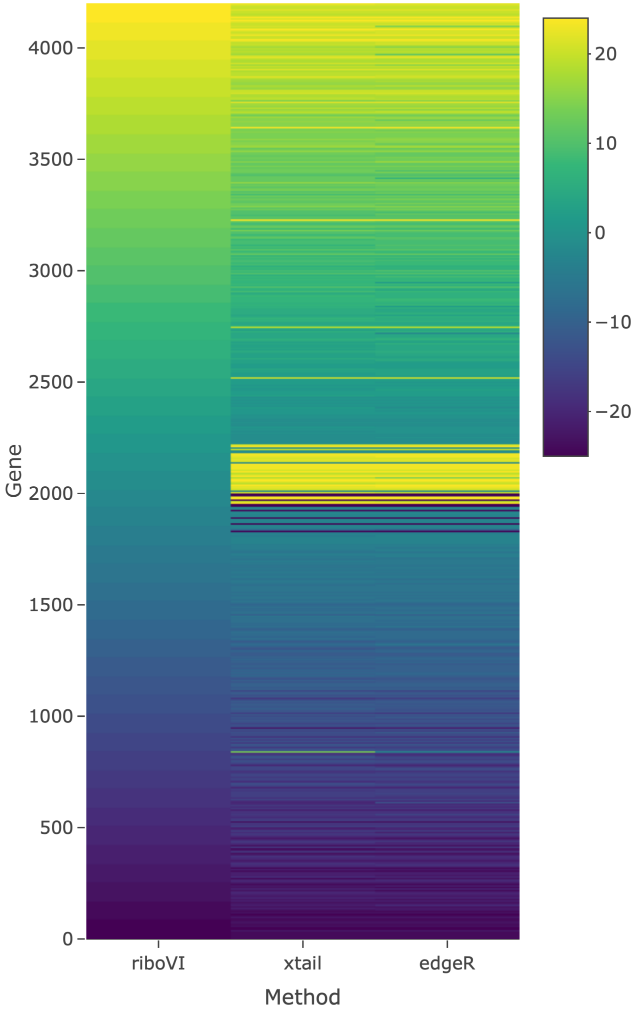

Figure S5. Heatmap of estimated translational regulation for each of 3 methods for genes flagged by `riboVI` at 5 DPI with nominal FDR = 0.05. Positive values (yellower colors) correspond to translational stimulation; negative values (bluer colors) correspond to translational repression.

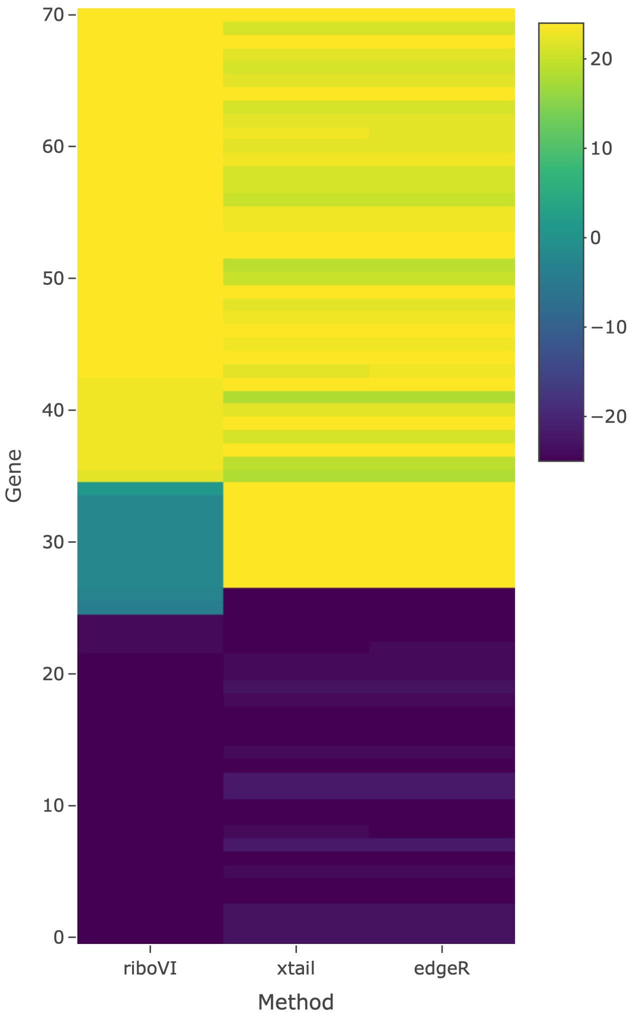

Figure S6. Heatmap of estimated translational regulation for each of 3 methods for genes flagged by `xtail` at 5 DPI with nominal FDR = 0.05. Positive values (yellower colors) correspond to translational stimulation; negative values (bluer colors) correspond to translational repression.

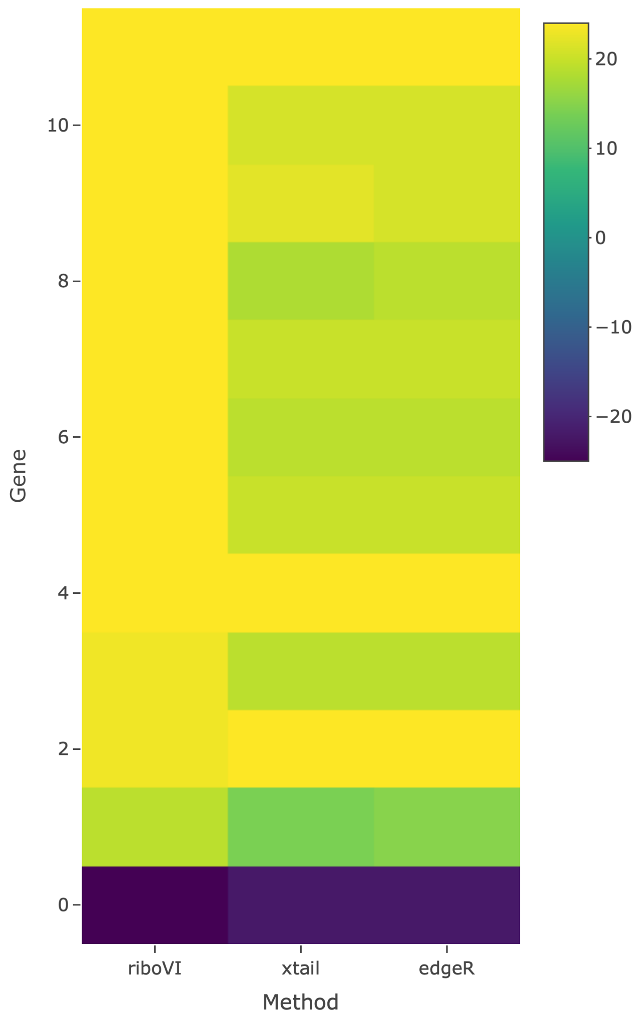

Figure S7. Heatmap of estimated translational regulation for each of 3 methods for genes flagged by `edgeR` at 5 DPI with nominal FDR = 0.05. Positive values (yellower colors) correspond to translational stimulation; negative values (bluer colors) correspond to translational repression.

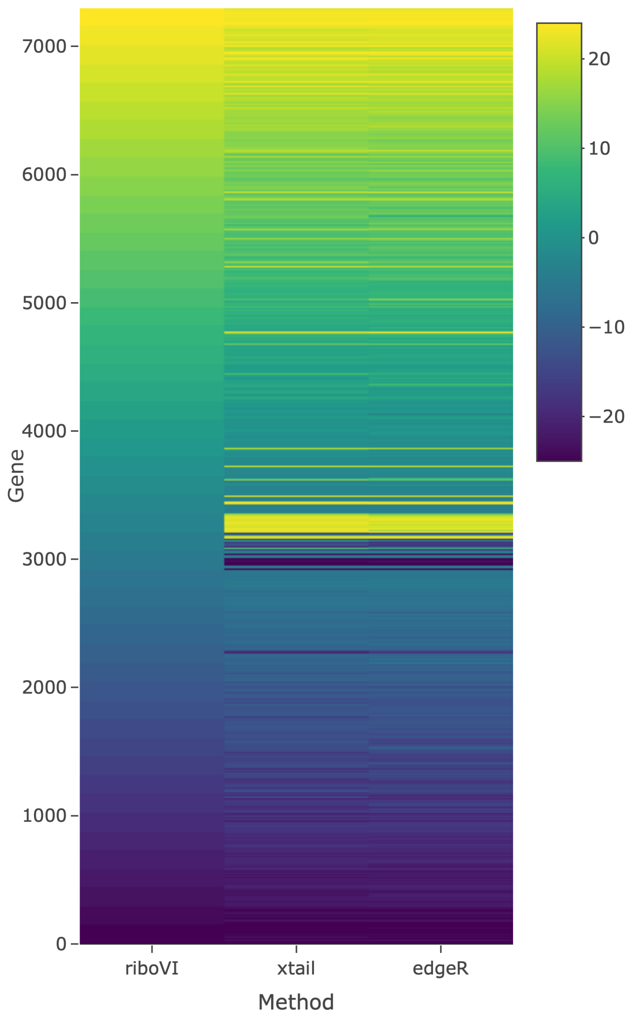

Figure S8. Heatmap of estimated translational regulation for each of 3 methods for genes flagged by `riboVI` at 8 DPI with nominal FDR = 0.05. Positive values (yellower colors) correspond to translational stimulation; negative values (bluer colors) correspond to translational repression.

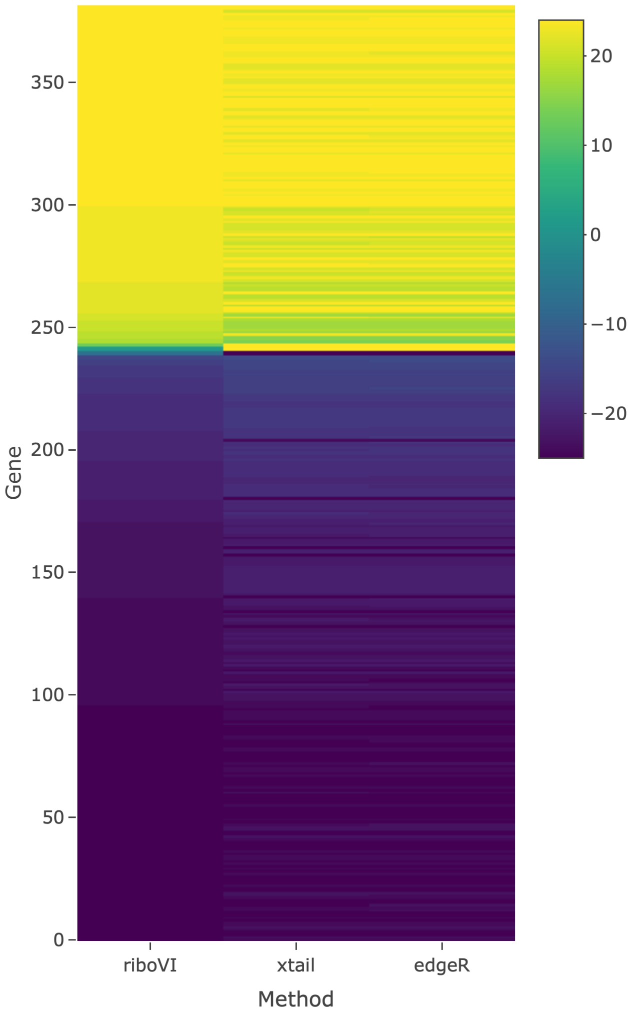

Figure S9. Heatmap of estimated translational regulation for each of 3 methods for genes flagged by `xtail` at 8 DPI with nominal FDR = 0.05. Positive values (yellower colors) correspond to translational stimulation; negative values (bluer colors) correspond to translational repression.

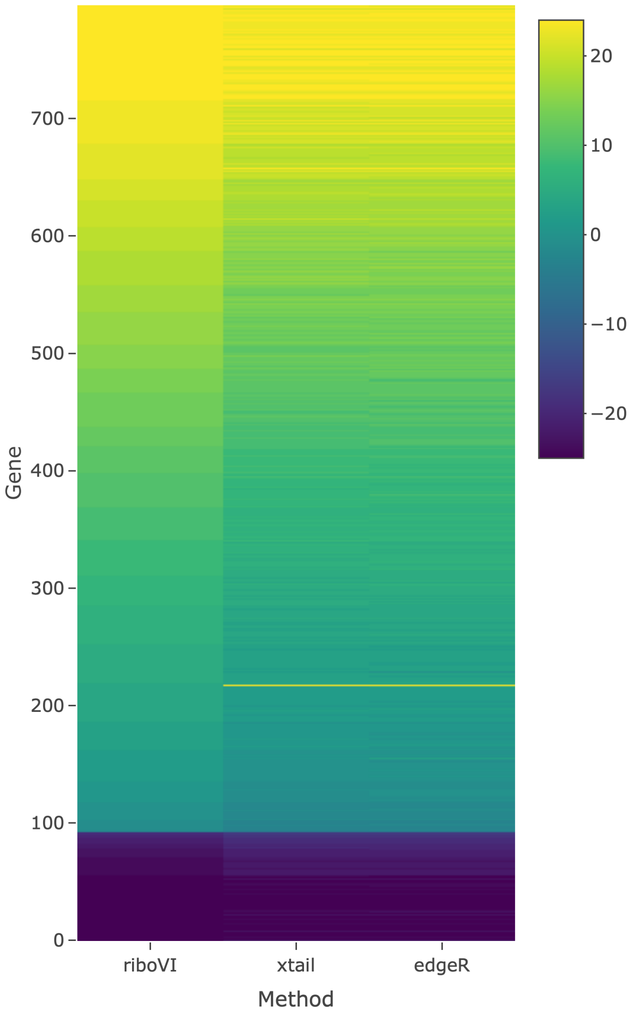

Figure S10. Heatmap of estimated translational regulation for each of 3 methods for genes flagged by `edgeR` at 8 DPI with nominal FDR = 0.05. Positive values (yellower colors) correspond to translational stimulation; negative values (bluer colors) correspond to translational repression.

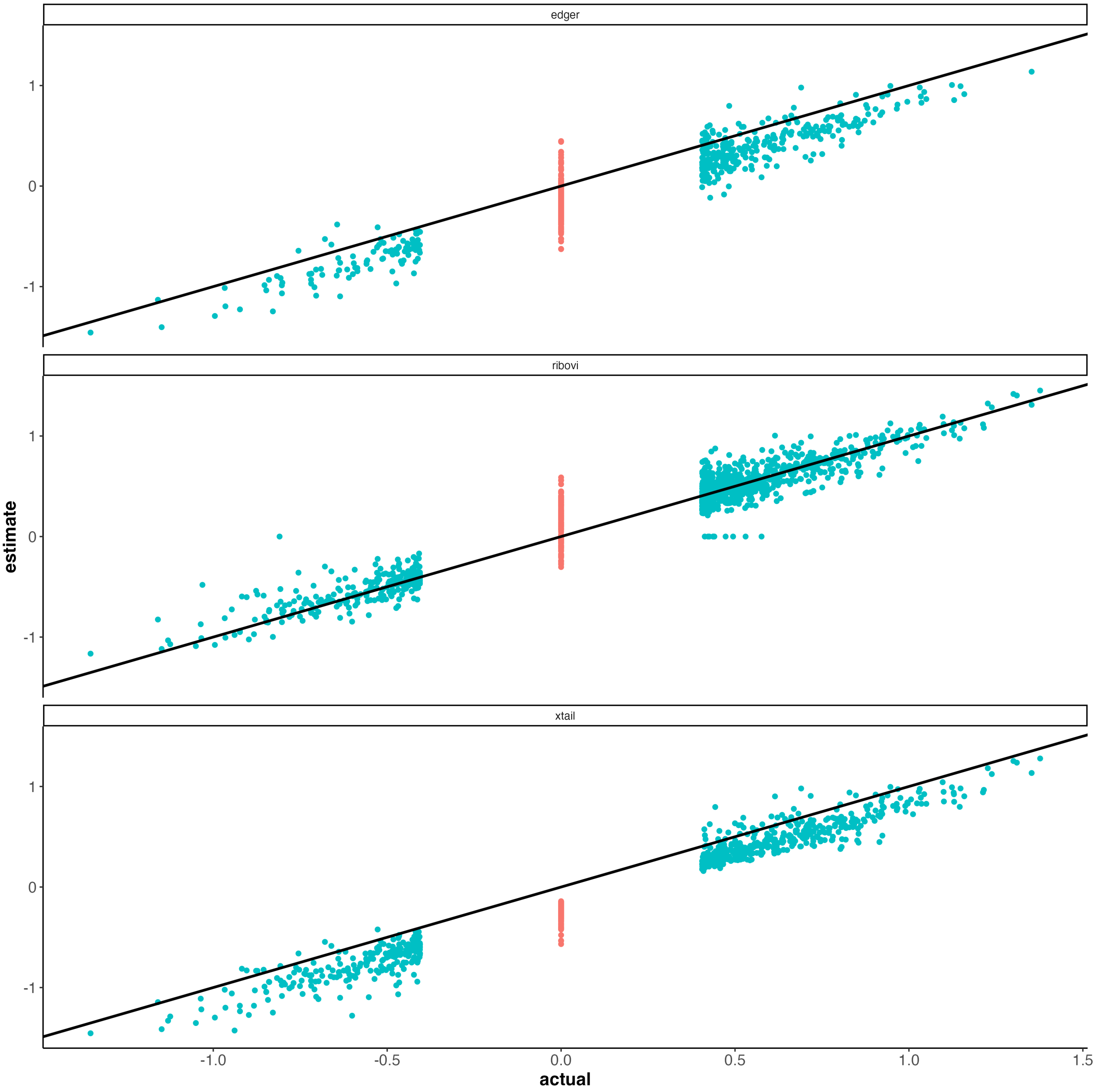

Figure S11. Simulated log TE change against model estimates for Simulation D, including only true positive (green) and false positive (red) genes for each method at nominal FDR = 0.05

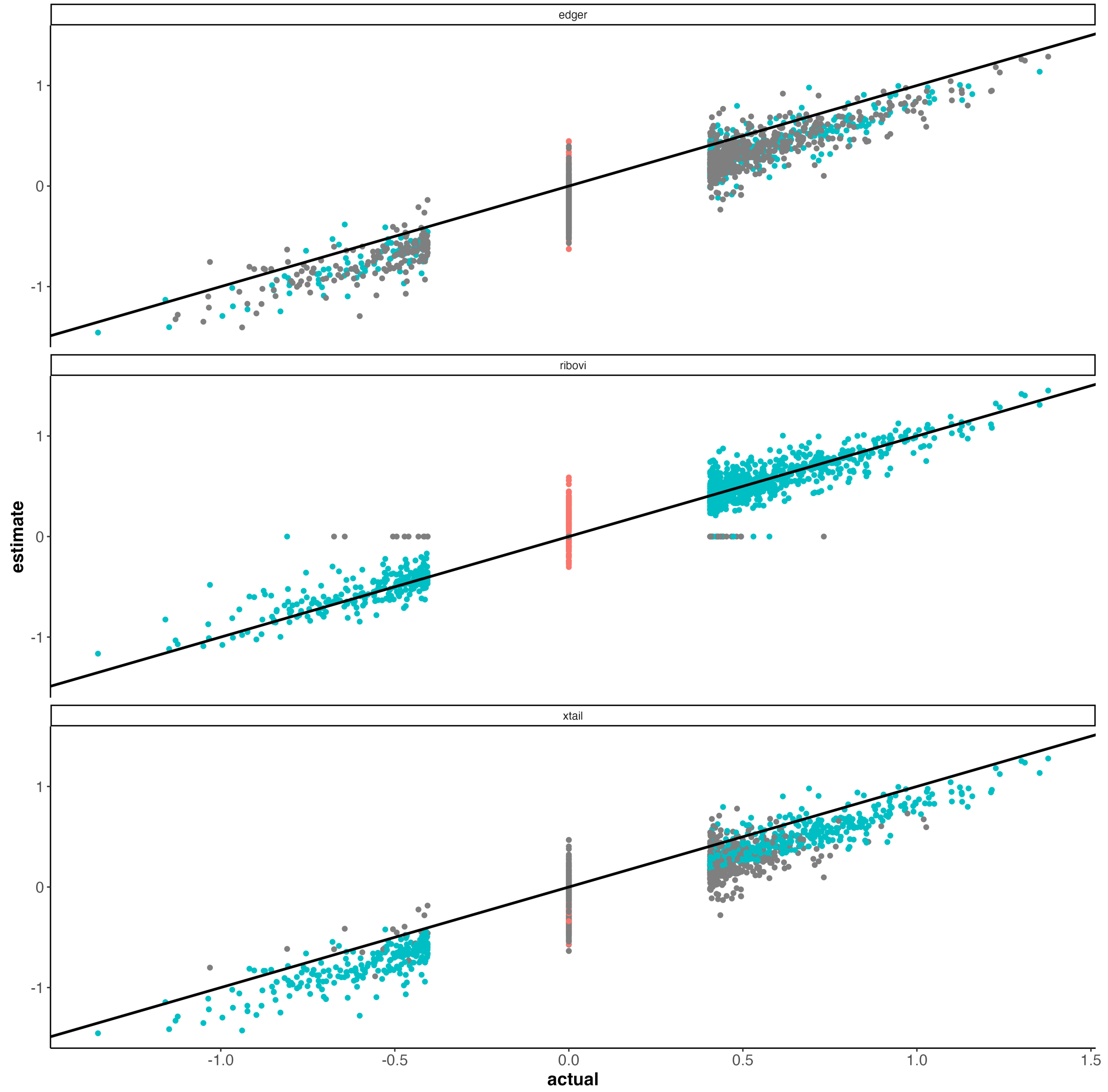

Figure S12. Simulated log TE change against model estimates for Simulation D, including true positive (green), false positive (red), and non-flagged (grey) genes for each method at nominal FDR = 0.05

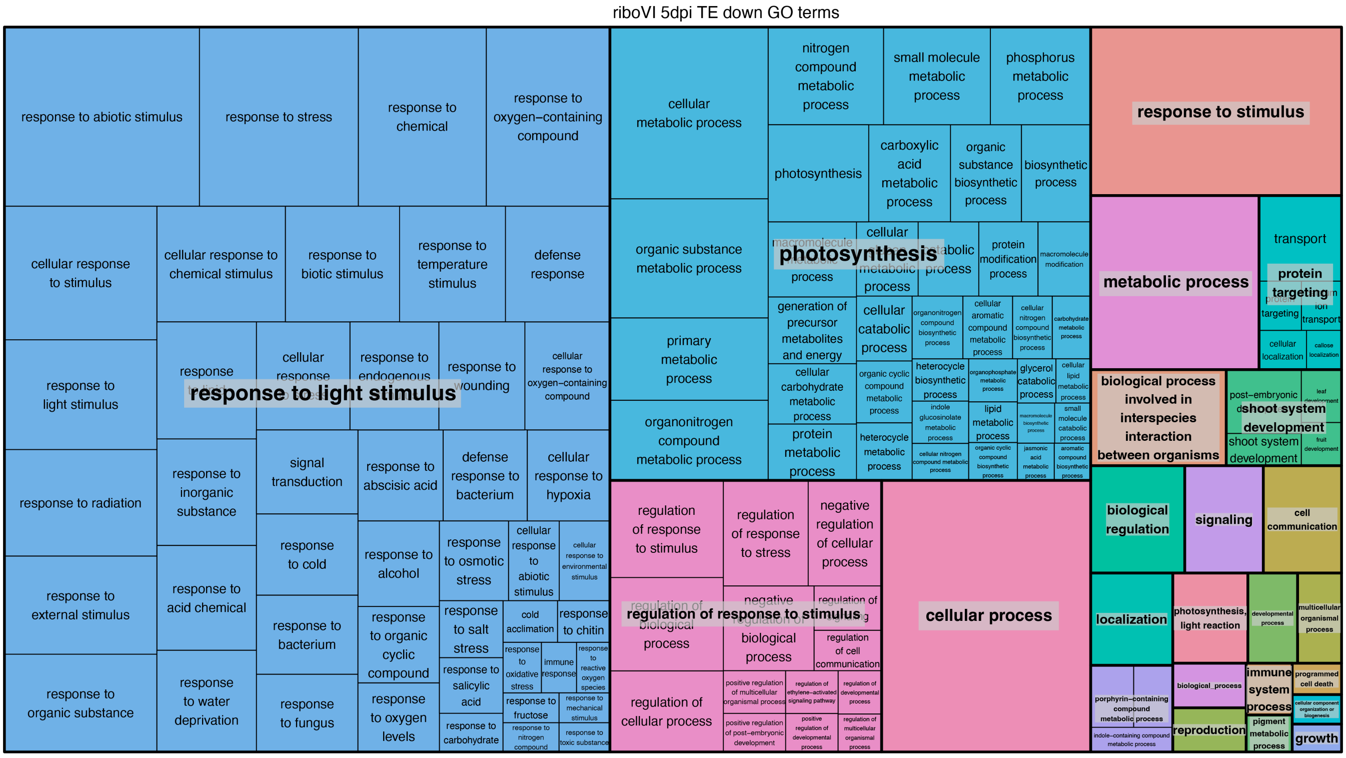

Figure S13. GO terms identified by `riboVI` at 5 DPI

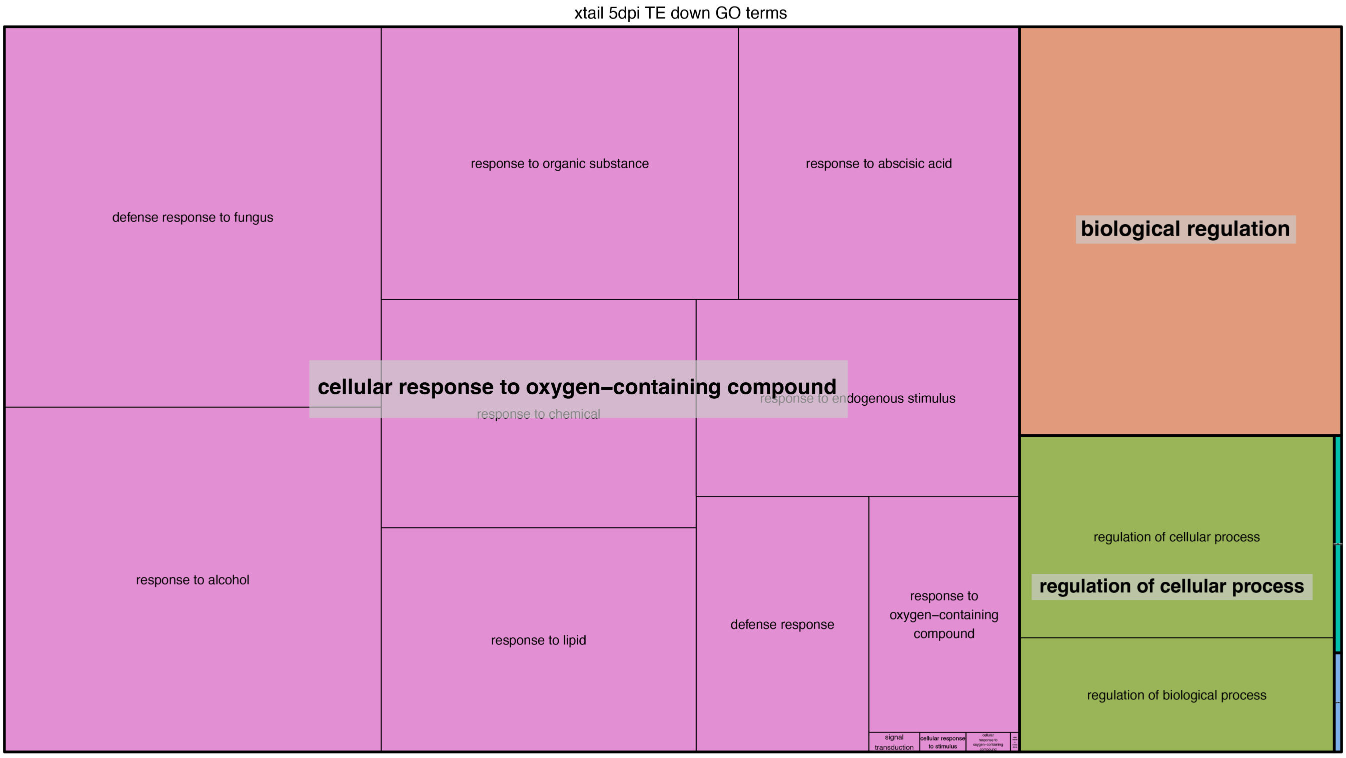

Figure S14. GO terms identified by `xtail` at 5 DPI
